# A genomics approach reveals insights into the importance of gene losses for mammalian adaptations

**DOI:** 10.1038/s41467-018-03667-1

**Published:** 2018-03-23

**Authors:** Virag Sharma, Nikolai Hecker, Juliana G. Roscito, Leo Foerster, Bjoern E. Langer, Michael Hiller

**Affiliations:** 1 Max Planck Institute of Molecular Cell Biology and Genetics, Pfotenhauerstr. 108, 01307 Dresden, Germany; 2 Max Planck Institute for the Physics of Complex Systems, Noethnitzer Str. 38, 01187 Dresden, Germany; 3 Center for Systems Biology Dresden, Pfotenhauerstr. 108, 01307 Dresden, Germany

**Keywords:** Genome informatics, Evolutionary genetics, Molecular evolution

## Abstract

Identifying the genomic changes that underlie phenotypic adaptations is a key challenge in evolutionary biology and genomics. Loss of protein-coding genes is one type of genomic change with the potential to affect phenotypic evolution. Here, we develop a genomics approach to accurately detect gene losses and investigate their importance for adaptive evolution in mammals. We discover a number of gene losses that likely contributed to morphological, physiological, and metabolic adaptations in aquatic and flying mammals. These gene losses shed light on possible molecular and cellular mechanisms that underlie these adaptive phenotypes. In addition, we show that gene loss events that occur as a consequence of relaxed selection following adaptation provide novel insights into species’ biology. Our results suggest that gene loss is an evolutionary mechanism for adaptation that may be more widespread than previously anticipated. Hence, investigating gene losses has great potential to reveal the genomic basis underlying macroevolutionary changes.

## Abstract (web summary)

Gene losses are generally considered detrimental, or at best neutral. Here, Sharma and colleagues present a new comparative genomics method to detect gene losses and highlight cases of gene losses in mammals that have potentially contributed to adaptive phenotypic innovations.

## Introduction

One of the most fascinating aspects of nature is the diversity of life. Mammals, for example, live in many different habitats, including land, air, and water, and exhibit remarkable phenotypic adaptations to their environment. A key challenge of contemporary biology is to understand the evolution of phenotypic diversity at the molecular level. This requires identifying the genetic origin of adaptive phenotypes, i.e., the involved genomic changes, which may reveal insights into the underlying molecular and cellular mechanisms. Numerous sequenced genomes have now made it possible to use comparative genomics to associate genomic differences with phenotypic differences between species^1–9^.

One genetic mechanism contributing to phenotypic differences is the inactivation (loss) of ancestral protein-coding genes^10,11^. In contrast to abundant pseudogenes that arose by duplication or retrotransposition^12^, gene loss (also known as a unitary pseudogene^13^) implies the absence of an intact gene encoding a functional protein, and thus affects the repertoire of gene functions. Case studies investigating the fate of selected genes uncovered associations between gene losses and several mammalian phenotypes^14–16^. These studies also revealed that gene loss in humans or human individuals can be adaptive by enhancing protection against pathogenic bacteria or diseases such as plasmodium and HIV infections, and sepsis^17–20^. In bacteria, laboratory selection experiments demonstrated that gene loss is a frequent cause of adaptations to various environmental conditions^21^. However, it is largely unknown whether gene loss could also play an important role for natural phenotypic adaptations in non-human mammals^11^.

To investigate the contribution of gene loss to phenotypic evolution, we develop a genomics approach to detect gene-inactivating mutations across many genomes at high accuracy. Using sequenced genomes of 62 placental mammals, we search for gene loss events that occurred specifically in mammals that exhibit prominent morphological, physiological, or metabolic adaptations. This reveals a number of previously unknown gene losses that are likely a consequence of adaptations or may contribute to adaptations that evolved in individual or even in multiple mammalian lineages. Our results suggest that gene loss is a mechanism that has likely contributed to adaptive evolution in several mammals.

## Results

### An approach to accurately detect gene loss events

To investigate the role of gene losses for phenotypic adaptations in mammals, a genomics approach to detect gene-inactivating mutations across many species and at high accuracy is required. Previous studies that comprehensively discovered and characterized genes that are lost in humans and related primates were limited to the human genome^19^ or involved manual curation^13,22^, which prevents a large-scale application to many other species. Therefore, we developed a computational approach to classify protein-coding genes as intact or lost. For a gene to be classified as lost, we require that a lineage, which descends from an ancestor with an intact gene, exhibits several gene-inactivating mutations that most likely result in a non-functional protein. As gene-inactivating mutations, we consider frameshifting insertions and deletions, in-frame stop codon mutations, and splice site-disrupting mutations. In addition, we consider the loss of exons or even entire genes, which could occur due to either large deletions in the genome or the accumulation of numerous mutations that destroy any sequence similarity. Our general approach is based on alignments between the genome of a reference species (here human), where a large set of genes is annotated, and the genomes of different query species (here 62 other mammals), where we search for inactivating mutations in these genes (Supplementary Fig. 1).

**Fig. 1:**
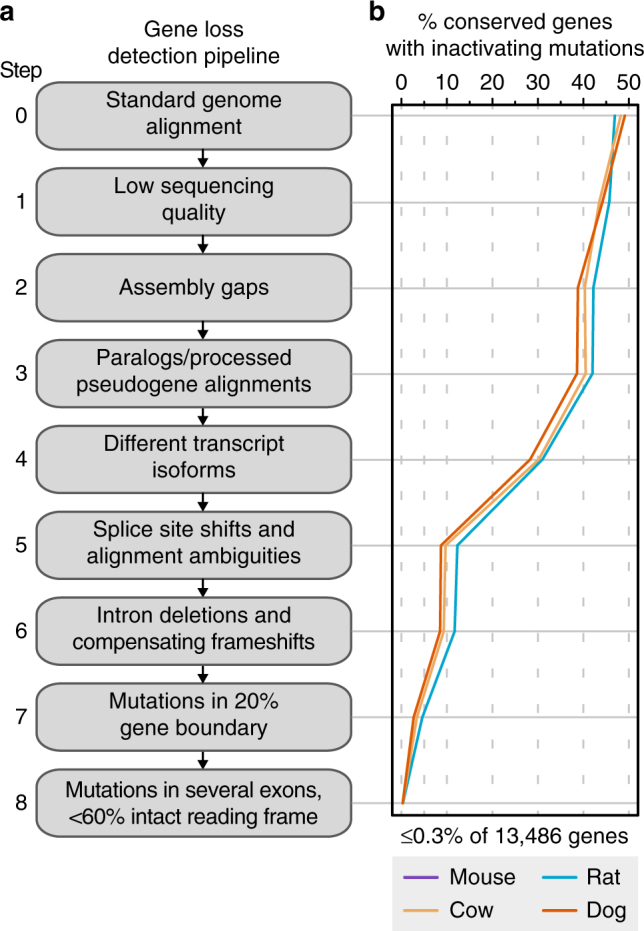
Genomics approach to detect gene loss events. **a** The different steps address a number of challenges related to assembly and alignment issues, and evolutionary changes in gene structures. **b** Applied to 13,486 human genes that have annotated 1:1 orthologs in mouse, rat, cow, and dog, these steps systematically reduce the number of conserved genes that have inactivating mutations. A total of 85–91% of the genes remaining after step 7 had inactivating mutations only in a single exon that is not entirely conserved (Supplementary Figs. 10 and 11). This shows that mutations in an individual exon of an otherwise-conserved gene is not sufficient to infer gene loss. By requiring that inactivating mutations occur in multiple exons and that less than 60% of the reading frame remains intact, our approach misclassifies ≤0.3% of 13,486 conserved genes as lost

Accurately detecting gene-inactivating mutations in these alignments poses a number of challenges. For example, sequencing errors and cases of assembly incompleteness (Supplementary Figs. 2 and 3), problems related to alignments (Supplementary Figs. 4–5), and evolutionary changes in the exon–intron structures of conserved genes (splice site shifts, lineage-specific exons, and precise intron deletions; Supplementary Figs. 5–7), all mimic inactivating mutations in genes that are in fact conserved. Furthermore, even real mutations may not indicate gene loss, for example when two frameshifting indels compensate each other (Supplementary Fig. 8) or when such mutations occur close to the N or C termini of the encoded proteins (Supplementary Fig. 9), which are under less evolutionary constraint^23,24^. To overcome these challenges and to achieve a high accuracy in detecting real gene-inactivating mutations, we implemented a series of filter steps (Fig. 1a). We tested our approach on a large set of 13,486 human genes that are conserved in mouse, rat, cow, and dog, and thus should not exhibit real inactivating mutations. The series of filters integrated in our approach drastically reduced the number of conserved genes with inactivating mutations such that gene loss is incorrectly inferred for ≤0.33% (32–45) of these 13,486 genes (Fig. 1b and Supplementary Table 1).

### Detecting gene loss events in placental mammals

We applied this approach to 62 placental mammals (Supplementary Table 2), which uncovered many known gene losses as well as numerous novel ones (Supplementary Table 3 and Supplementary Fig. 12). To investigate the contribution of gene loss to adaptive evolution, we used this data to search for genes that are specifically lost in lineages that exhibit prominent phenotypic adaptations, while being intact in other species that do not share these adaptations. For all previously unknown gene losses presented here, we further confirmed the loss by validating the gene-inactivating mutations with unassembled sequencing reads from the respective species.

Based on the following rationale, we distinguished between gene losses that may have contributed to an adaptation and those that are likely a consequence of the evolution of an adaptive phenotype. If a gene loss event contributed to an adaptation, we expect that loss-of-function mutations introduced in a related model organism result in phenotypes that are highly similar to the naturally occurring adaptive phenotype and that the age of the gene loss coincides with a period during which this adaptation evolved. In contrast, if the gene loss event is a consequence of relaxed selection following an adaptation, we expect that knockout phenotypes and molecular function do not have a causal relationship to the adaptive phenotype or that the gene was lost after the evolution of this phenotype. By making use of existing gene knockouts in mouse or loss-of-function mutations in human individuals and by dating gene loss events, we discovered a number of previously unknown gene losses (Supplementary Fig. 13 and Supplementary Table 4), some of which may have contributed to morphological, physiological, and metabolic adaptations in mammals, while others are likely a consequence of adaptive evolution.

### Epidermal adaptations in cetaceans

**Fig. 2:**
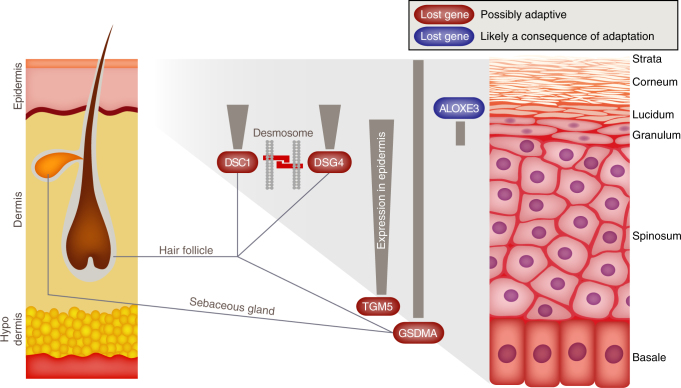
Adaptations of the cetacean epidermis to the aquatic environment. The figure shows genes with hair- and epidermis-related functions that are specifically lost in cetaceans. The expression pattern of these genes in the skin is shown as gray lines and boxes (expression gradients are indicated). Mice in which these genes are knocked out show epidermal phenotypes that strongly resemble morphological adaptations of the cetacean skin. Since the loss of *DSG4*, *DSC1*, *TGM5*, and *GSDMA* coincided with a period during which epidermal adaptations evolved in cetaceans (Supplementary Note 1, Supplementary Figs. 14–17, and Supplementary Table 5), these gene losses could have played a causal role in the remodeling of cetacean epidermis. The cetacean-specific loss of *ALOXE3*, an atypical lipoxygenase that is important for skin barrier function, happened after the split of the baleen and toothed whale lineage (Supplementary Fig. 18 and Supplementary Table 5) and is thus presumably a consequence of epidermal adaptations in these lineages

We first focused on the unique skin morphology of cetaceans, which is highly adapted to the aquatic environment by exhibiting (i) a much thicker epidermis that enhances physical barrier properties and protects against the greater pressure in their much denser environment^25^, (ii) a high shedding rate of cells in the stratum corneum that maintains a smooth surface and limits microbe colonization^26^, and (iii) no hair to reduce drag while swimming^27^. Our analysis revealed genes with hair- and epidermis-related functions that are specifically lost in all four cetaceans present in our analysis (dolphin, orca, sperm whale, minke whale) (Fig. 2; all genes, their inactivating mutations and loss date estimations are described in detail in Supplementary Note 1, Supplementary Figs. 14–17, and Supplementary Table 5). Mice in which *GSDMA*, *DSG4*, or *DSC1* are knocked out exhibit key aspects of the cetacean skin morphology, in particular a thicker epidermis and loss of hair. Furthermore, *DSG4* and *DSC1* encode components of desmosomes that mediate cell adhesion in the upper epidermis, and cetaceans also lost the peeling skin syndrome gene *TGM5* that cross-links structural corneocyte proteins. The loss of these three genes suggests that fewer desmosomes and impaired protein cross-links could be a mechanistic explanation for the high shedding rate of stratum corneum cells. Since the loss of these genes predated or coincided with the split of the cetacean ancestor (Supplementary Note 1, Supplementary Figs. 14–17, and Supplementary Table 5), these gene losses could have contributed to the remodeling of the cetacean epidermis morphology.

### Diving and dietary adaptations in sperm whales

**Fig. 3:**
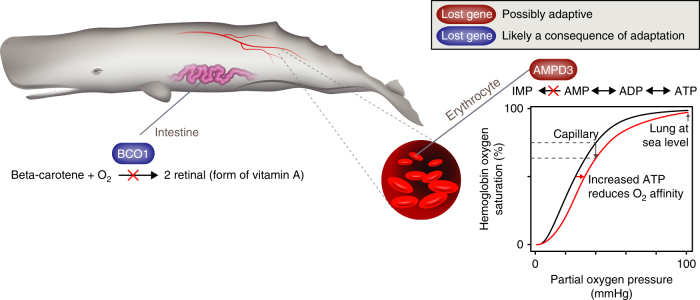
Diving and dietary adaptations in sperm whales. The loss of *AMPD3* (red, Supplementary Fig. 19) is likely an adaptation to the extreme diving ability of sperm whales. *AMPD3* deaminates adenosine monophosphate (AMP) to inosine monophosphate (IMP) in erythrocytes. *AMPD3* loss increases the level of ATP (an allosteric hemoglobin effector), which facilitates O_2_ release, as illustrated by the O_2_-hemoglobin dissociation curve (wildtype, black; *AMPD3* knockout, red^28^). In contrast, the loss of the vitamin A synthesizing enzyme *BCO1* (blue, Supplementary Fig. 20) in sperm whales is likely a consequence of relaxed selection after sperm whales adapted to their specialized diet that mainly consists of vitamin A-rich but beta-carotene poor squid. The absence of its substrate (beta-carotene) likely made this enzyme obsolete, leading to the loss of *BCO1*

To explore if gene losses can be causally involved in physiological adaptations, we identified and examined genes that are specifically lost in the sperm whale, which is one of the deepest and longest diving mammals that routinely dives for 40–60 min to depths of >400 m. We detected the loss of *AMPD3*, estimated to have happened soon after the sperm whale lineage split from other toothed whales (Supplementary Note 2, Supplementary Fig. 19, and Supplementary Table 5). *AMPD3* encodes an erythrocyte-specific enzyme whose knockout in mice increases the erythrocyte ATP level by threefold^28^. Since ATP is an allosteric effector that stabilizes O_2_-unloaded hemoglobin in vertebrates, the loss of *AMPD3* results in a reduced O_2_ affinity of hemoglobin^28^. This is probably adaptive for long-diving sperm whales as a lower affinity facilitates O_2_ release from hemoglobin to the O_2_-depleted tissue (Fig. 3 and Supplementary Note 2). Remarkably, crocodiles that can stay submerged for over an hour also show a reduced O_2_ affinity of hemoglobin; however, this reduction is mediated by bicarbonate ions (HCO_3_^−^) instead of ATP^29^. Thus, the loss of *AMPD3* could be a novel adaptive mechanism to improve O_2_ transport from blood to tissue in a long-diving mammal.

We also detected another sperm whale-specific gene loss that is likely a consequence of a dietary specialization. Sperm whales feed predominantly on squid that contain no or very little beta-carotene, but are rich in vitamin A. This likely explains why the sperm whale is the only mammal in our data set that has lost the *BCO1* gene, which encodes an enzyme that cleaves beta-carotene into retinal (a form of vitamin A) (Fig. 3, Supplementary Note 3, and Supplementary Fig. 20). Since the loss of *BCO1* is unlikely to be causally involved in the evolution of a diet rich in squid, its loss is probably a consequence of relaxed selection after this dietary specialization evolved in the sperm whale. Together, the loss of *AMPD3* and *BCO1* in sperm whales suggest that gene losses can be both causally involved in adaptations and can be a consequence of relaxed selection after adaptations.

### Physiological and metabolic adaptations in fruit bats

**Fig. 4:**
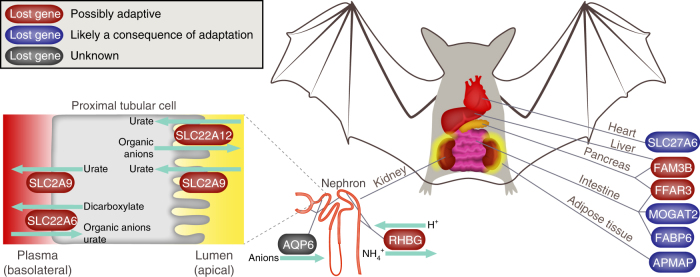
Renal and metabolic adaptations in frugivorous bats. A number of renal transporter genes (red, left side) that are specifically lost in fruit bats (large and black flying foxes) reduce urine osmolality in a mouse knockout. Thus, these gene losses likely contribute to the ability of fruit bats to efficiently excrete excess dietary water. Losses of metabolic genes (red, right side) are likely adaptive by improving the processing of the sugar-rich fruit juice. In contrast, gene losses shown in blue are probably a consequence of adapting to the frugivorous diet. These genes provide new insights into the metabolism of bats and corroborate the strong dependence of internal organs on using sugar as the main energy source. Please see Supplementary Figs. 21–31 for the inactivating mutations in these 11 genes

To investigate if gene loss is not only a consequence, but can also contribute to evolutionary adaptations to a highly specialized diet, we identified genes that are specifically lost in the ancestor of the large and black flying fox (Supplementary Notes 4 and 5, and Supplementary Figs. 21–31). These fruit bats feed predominantly on large amounts of juice extracted from fruits. This diet poses the challenge of excreting excess dietary water while preserving scarce electrolytes, which bats address by producing a very dilute urine^30^. Our results indicate that this ability is likely facilitated by the loss of *SLC22A12* (URAT1), *SLC2A9* (GLUT9), and *SLC22A6* (OAT1), three renal transporter genes whose knockout in mice reduces urine osmolality^31,32^ (Fig. 4 and Supplementary Note 4). The loss of *RHBG*, a renal ammonium secreting transporter, may be important for another renal adaptation to the frugivorous diet that contains abundant potassium but little sodium. Since ammonium inhibits potassium secretion and sodium reabsorption^33^, the loss of the ammonium secreting *RHBG* may contribute to the ability of fruit bats to efficiently excrete excess potassium and efficiently reabsorb scarce sodium^34^. Finally, the loss of poorly characterized kidney genes such as *AQP6*, the only aquaporin family member that functions as an anion-channel instead of a water channel^35^, indicates that additional gene losses could be related to kidney adaptations in fruit bats. Together, these gene losses suggest that the simplification of the renal transporter repertoire is an evolutionary mechanism that contributes to the ability of fruit bats to excrete excess dietary water while preserving electrolytes.

The second challenge faced by fruit bats is the nutritional composition of their specialized diet, consisting predominantly of sugars and very little fat and protein^36^. We identified the loss of two genes involved in insulin metabolism and signaling (*FFAR3* and *FAM3B*) that is likely adaptive by improving metabolic processing of ingested sugar (Fig. 4 and Supplementary Note 5). The loss of *FFAR3*, encoding an insulin secretion inhibitor^37^, may explain why fruit bats secrete substantially more insulin than other mammals^38^. The loss of *FAM3B*, encoding a cytokine that is co-secreted with insulin from pancreatic beta-cells^39^, may contribute to enhanced hepatic insulin sensitivity, as observed in *FAM3B* knockout mice^40^. In addition, other gene losses are presumably a consequence of relaxed selection after adapting to the frugivorous diet (Fig. 4 and Supplementary Note 5) and reveal hitherto unknown metabolic aspects of how different organs adapted to using sugar as the major energy source. For example, the loss of FATP6 (*SLC27A6*) that transports fatty acids into cardiac myocytes^41^ indicates that sugars replace fatty acids as the major energy source in the heart. The loss of *MOGAT2* and *FABP6*, two genes involved in intestinal fat digestion, is likely also a consequence of the fat-poor frugivorous diet. Finally, the loss of *APMAP*, a gene required for adipocyte differentiation^42^, is likely related to the small size and rapid turnover of fat depots in fruit bats, which reflect the energetically costly process of converting sugars into fat^43,44^. In summary, several fruit bat-specific gene losses reveal new insights into the metabolism of fruit bats and suggest that gene loss could be a genetic mechanism involved in metabolic and physiological adaptations to a frugivorous diet.

Overall, our analysis shows that several gene losses may have contributed to or are a consequence of different types (morphological, physiological, and metabolic) of adaptations in different mammalian lineages, suggesting that gene losses play an integral role in phenotypic evolution in mammals.

### Convergent gene loss and repeated phenotypic adaptations

**Fig. 5:**
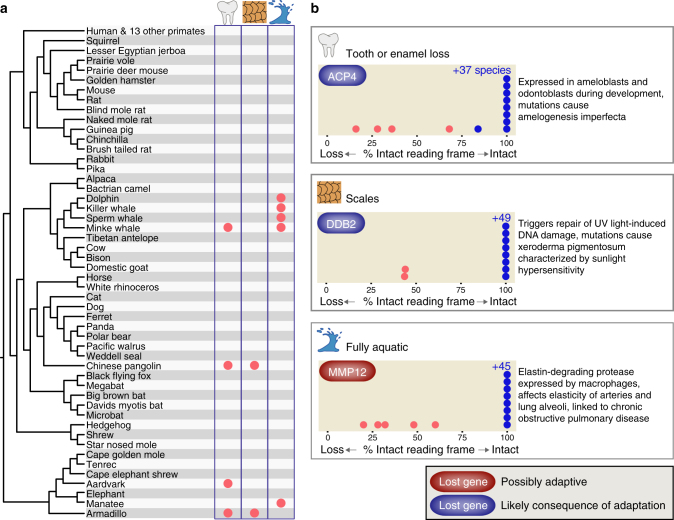
Convergent gene losses and repeated phenotypic adaptations. **a** Phylogenetic tree showing the independent lineages that share a derived phenotype (loss of teeth or enamel, body armor in the form of scales, fully aquatic lifestyle). **b** A new Forward Genomics method discovered genes that are preferentially lost in mammals with the derived phenotype (red dots) compared to other mammals (blue dots). The panel shows the maximum percentage of the intact reading frame of these genes in each species and summarizes gene function (see also main text, Supplementary Notes 6–8 and Supplementary Figs. 33–35). *ACP4* has a %intact reading frame value of 86% in the Tibetan antelope (blue dot); however, no unassembled sequencing reads are available to validate the mutation. For some species, we could not compute the maximum intact reading frame due to missing data, therefore the total number of evaluated species slightly differs per gene (Supplementary Tables 6–8)

If gene loss is an important evolutionary mechanism for phenotypic change, we expect that convergent gene loss is also a predictable consequence and may contribute to similar adaptations that independently evolved in multiple lineages. To search for convergent gene losses, we adopted the previously developed “Forward Genomics” framework^6,9^ to detect correlations between the maximum percentage of the intact reading frame and independently evolved adaptations (Supplementary Fig. 32). Our approach utilizes phylogenetic generalized least squares^45^ to correct for phylogenetic relatedness between mammals. As a proof of concept, we searched for genes that are lost in four mammals that independently lost tooth enamel (Fig. 5a) and identified previously known losses of the tooth-specific genes *MMP20* and *C4orf26*^15^ that are essential for enamel formation (Fig. 5b, Supplementary Note 6, and Supplementary Table 6). As a novel result, we detected the convergent loss of *ACP4* (Fig. 5b and Supplementary Fig. 33), a gene that is associated with the enamel disorder amelogenesis imperfecta^46^. This shows that a genome-wide search can identify known and novel gene losses that are involved in the independent loss of enamel.

Next, we searched for gene losses specific to armadillo and pangolin, two mammals that independently evolved body armor in the form of scales. As the scales of these two species have different developmental origins (made of keratin in pangolins and bone in armadillos), it is unlikely to find gene losses that play a causal role in scale formation; instead, forward genomics may identify genes that are lost as a consequence of scale evolution, which could reveal unknown aspects related to body armor. Surprisingly, we found that both scaly mammals are the only species in our data set that have lost the *DDB2* gene (Fig. 5, Supplementary Note 7, Supplementary Fig. 34, and Supplementary Table 7). DDB2 detects pyrimidine dimers caused by UV light and triggers nucleotide excision DNA repair^47^. Mutations in human and mouse *DDB2* compromise DNA repair and cause xeroderma pigmentosum^48^, a disease characterized by hypersensitivity to sunlight. Thus, the convergent loss of *DDB2* in mammals whose sun-exposed, dorsal skin is covered by scales suggests the possibility that scales are sufficient to protect from UV light-induced DNA damage. While *DDB2* loss in armadillo, a lineage where the timing of scale evolution is not well understood, appears to be relatively old, the gene loss in pangolin most likely happened after the evolution of scales^49^ (Supplementary Table 5), which suggests that *DDB2* loss is a consequence of body armor evolution in that lineage.

We further explored whether loss of the same gene could contribute to similar phenotypic adaptations shared between two lineages. Searching for gene losses shared between the fully aquatic cetacean and manatee lineages revealed *KLK8*, a gene loss that correlates with skin and neuroanatomical differences of aquatic mammals^50^. In addition, we discovered the loss of *MMP12* (Fig. 5, Supplementary Note 8, Supplementary Fig. 35, and Supplementary Table 8), which provides the first insights into the molecular mechanism underlying a unique breathing adaptation. The so-called “explosive exhalation” allows cetaceans and manatees to renew ~90% of the air in a single breath^51^, and is advantageous for fully aquatic mammals by clearing the blowhole/nostrils before inhaling and by minimizing time at the surface, where wave drag slows swimming^52^. Explosive air exchange is facilitated by extensive elastic tissue in the lungs that permits a greater expansion during inhalation and whose elastic recoil helps to empty the lungs quickly^53^. *MMP12* encodes a potent protease that degrades elastin, the major component of elastic fibers^54^. Hyperactivity of *MMP12* in the lung can be induced by cigarette smoke in humans and reduces the elasticity of alveoli, which contributes to an incomplete emptying of the lung in chronic obstructive pulmonary disease patients^55^. Thus, the specific loss of the elastin degrading *MMP12* suggests a mechanism that could contribute to the extensive elastic lung tissue necessary for explosive exhalation. Supporting this, *MMP12* loss predates the split of the fully aquatic toothed and baleen whale lineages and happened early in the fully aquatic sirenia (manatee) lineage (Supplementary Table 5). Overall, by discovering novel associations between convergent gene losses and independently evolved phenotypic adaptations, we provide additional evidence that gene losses are not only a consequence, but can be a repeated mechanism for similar phenotypic adaptations.

## Discussion

In this study, we showed that evolutionary gene losses are not only a consequence, but may also be causally involved in phenotypic adaptations. Gene loss as a consequence of adaptation is likely the result of relaxed selection to maintain a gene whose function became obsolete. This “use it or lose it” principle could explain the loss of enzymes whose substrate is scarcely available (sperm whale *BCO1*), the loss of genes involved in fat digestion in species with a fat-poor diet (fruit bat *MOGAT2* and *FABP6*), and the loss of the amelogenesis-involved *ACP4* in enamel-less species. Importantly, gene losses as a consequence of adaptation can reveal unknown aspects related to the adaptation. For example, the loss of the cardiac fatty acid transporter *SLC27A6* suggests that the heart of fruit bats is highly dependent on sugar as the main energy source. Likewise, the loss of *DDB2* in scaly mammals suggests the possibility that their body armor sufficiently protects the animal against UV light-induced DNA damage.

Even though one would intuitively expect that loss of ancestral genes is typically maladaptive, gene loss can be beneficial by providing an evolutionary mechanism for phenotypic adaptations^11^. This “less is more” principle^56^ likely applies to the loss of genes involved in insulin signaling (*FAM3B* and *FFAR3*) and renal function (*SLC22A12*, *SLC22A6*, *SLC2A9*, and *RHBG*) in fruit bats, the loss of *AMPD3* that likely improves O_2_ transport in sperm whales, and the loss *MMP12* that may contribute to explosive exhalation in aquatic mammals. Moreover, the loss of epidermis-related genes (*DSG4*, *DSC1*, *TGM5*, and *GSDMA*) in cetaceans suggests that the loss of genes with specific functions and restricted expression patterns can also contribute to morphological adaptations. In summary, our study provides evidence that the loss of ancestral genes is not only a predictable consequence of phenotypic evolution, but may also be a driver for a variety of adaptations in mammals. More research is necessary to validate the hypothesis that adaptation by gene loss is not only an evolutionary mechanism for bacteria, but also for complex multicellular organisms.

If the loss of an existing (ancestral) gene would increase fitness by making a species better adapted to its environment, then gene loss is an easy solution to an evolutionary problem, because coding genes provide numerous positions for random mutations to inactivate them. While offering a relatively easy solution, gene loss is likely irreversible after several inactivating mutations accumulated. This irreversibility may indicate that gene function diversity is preferentially preserved in generalist species and that gene loss could influence macroevolutionary trajectories by hampering phenotypic reversal in highly adapted specialists. For example, if genes specifically lost in fruit bats are required for various diets, then fruit bats as dietary specialists might be limited in their ability to return to a generalist state by adapting to a different diet. This may not only have implications for conservation efforts, but also offers the testable hypothesis that gene loss, both as a consequence and cause of specialization, could explain why some specialist lineages have a limited capacity to persist and diversify over macroevolutionary timescales^57^.

Our study highlights the power of comparative genomics to reveal insights into the genomic basis of complex adaptive phenotypes. Our approach has broad applicability to generate high-quality gene loss catalogs for the forthcoming “flood of genomes”, of both mammals and other species. The presented gene loss-based forward genomics approach can be applied to detect new associations between gene losses and convergent phenotypes. Growing resources of well-characterized gene knockouts in mouse^58^ and other model organisms will further help to discover novel gene losses that are potential causes or consequences of phenotypic evolution. Hence, the concept of adaptation by gene loss has great potential to uncover the molecular mechanisms underlying the evolution of a wide range of adaptive phenotypes, which will deepen our understanding of how nature’s fascinating phenotypic diversity has evolved.

## Methods

### Genomics approach to detect gene loss events

To detect intact genes and lost genes (also called unitary pseudogenes^13^), we made use of a whole-genome alignment between human (hg38 assembly) and placental mammals. These alignments were obtained with parameters (lastz^59^ parameters *K* = 2400 and *L* = 3000) that are sufficiently sensitive to align exons among placental mammals^60^. Genome alignments are more appropriate for detecting gene loss events than existing gene annotations, since the absence of an annotation for a gene can also be due to incomplete genomic data or other artifacts. Furthermore, gene annotations are not available for many placental mammals. Therefore, we used the gene annotation of a reference species (here, Ensembl version 90 for the human hg38 genome) and investigated the potential loss of these 19,425 genes by searching the genome alignment for gene-inactivating mutations in 62 placental mammals (Supplementary Table 2). Since placental mammals are separated by an evolutionary distance of ~0.5 or fewer substitutions per neutral site^61^, we did not only search for the complete loss of exons or entire genes, but also searched for the following gene-inactivating mutations: (i) insertions and deletions that shift the reading frame, (ii) frame-preserving insertions (for example due to a transposon insertion) that create a premature stop codon, (iii) substitutions that create an in-frame stop codon, and (iv) mutations that disrupt splice sites. We considered a disrupted splice site as a deviation from the consensus donor splice site (GT/GC) or the consensus acceptor splice site (AG). Since big insertions or deletions (indels) are rare in conserved genes, we also considered frame-preserving indels longer than 50 bp as an inactivating mutation.

To exclude potential artifacts that can mimic real gene-inactivating mutations, we employed a series of filters. First, we excluded deleted or unaligning exons or genes from the list of inactivating mutations if the respective genomic region (defined by the nearest up- and downstream aligning blocks) overlaps an assembly gap in the query species. Second, we only considered genes that occur in a context of conserved gene order in a query species to exclude potential mis-alignments to processed pseudogenes and paralogs that are typically located in a different context. This also implies that all considered exon or gene deletions occur in an otherwise-conserved context. Third, to avoid cases where an inactivating mutation is not observed in an alternative exon alignment, we re-aligned each coding exon with CESAR^24,62^ (default parameters). CESAR is a Hidden–Markov model-based exon aligner that takes splice site and reading frame information into consideration and finds an intact exon alignment whenever possible. Only inactivating mutations that were observed in the CESAR alignment were further considered.

Furthermore, since we used human gene annotations as a starting point, we employed additional filters to exclude cases where exon–intron structures of conserved genes have changed in evolution. First, we considered all principal or alternative isoforms from the APPRIS database^63^, which provides those isoforms of a given gene that exhibit the highest cross-species conservation and the most conserved protein features. For each query species, we then considered the isoform with the lowest number of inactivating mutations. Second, the CESAR re-alignment step detects cases where the position of splice sites has shifted^62^. CESAR also explicitly considers the possibility of precise intron deletions, which simply result in a larger composite exon. In case of splice site shifts or intron deletions, we excluded the respective splice site mutations from the list of inactivating mutations. Importantly, since CESAR performs a pairwise exon alignment, it captures exon–intron structure changes that happened in either the human (reference) or the query lineage. Finally, since N or C termini of proteins are generally less constrained in evolution^23,24^, we removed all mutations that are within the first or last 20% of the protein sequence from the list of inactivating mutations. Together, these measures avoid assembly and alignment issues, and address evolutionary exon–intron structure changes in genes that are conserved. The resulting list of gene-inactivating mutations was used to determine the maximum percentage of the intact reading frame (%intact) for each gene and each query species. For example, if a gene has inactivating mutations at the relative positions 20% and 55% of the coding sequence, the %intact value is 45%. Apart from using %intact values with forward genomics (below), we considered genes as loss candidates if %intact is <60% and if at least 20% of the exons have inactivating mutations (for single-exon transcripts, we simply required at least two inactivating mutations).

Since the human Ensembl gene annotation includes genes that arose after the split of the placental ancestor and since loss of a gene in a query species requires that this gene should have been present in the common ancestor of human and query, we inferred the most ancient ancestor, where the gene was intact from all those query species that have no gene-inactivating mutations. Only gene loss events in species that descended from that ancestor were further considered.

To test the error rate of the approach, we applied it to a set of genes that are conserved between human and mouse/rat/dog/cow. To this end, we considered 13,486 genes that are annotated in these genomes and have a 1:1 orthologous relationship (downloaded from Ensembl Biomart^64^) to a human gene (Supplementary Data 1). After each step in our pipeline (Fig. 1), we determined the number of inactivating mutations and the number exons/genes with at least one such mutation (Supplementary Table 1).

To further ensure that all gene loss events discussed in this study (Supplementary Table 4) are real and not due to sequencing errors, we validated stop codon mutations, splice site mutations, and frameshifting insertions/deletions with unassembled sequencing reads stored in the sequence read archive (SRA)^65^ or the NCBI trace archive. To this end, we extracted the genomic context comprising at least 50 bp around the mutation and only kept those where at least 10 unassembled raw reads with exact matches provide conclusive evidence for the presence of the mutation, as previously described^50^. Notably, many of the reported genes have gene-inactivating mutations that are shared between two or more independently assembled species (such as four cetaceans or two fruit bats), which further supports that these gene-inactivating mutations are real.

### Investigating the role of gene loss for mammalian adaptations

To discover associations between lost genes and phenotypic adaptations, we first focused on those gene losses that are specific to certain mammalian lineages. Then, we integrated these gene losses with different functional annotations to single out genes that could be related to a phenotypic adaptation that evolved in these lineages. We used functional annotations from gene ontology^66^, mouse knockout phenotypes from mouse genome informatics (MGI)^67,68^, human phenotypic associations from the human phenotype ontology^69^ and OMIM^70^, and protein domain information from Interpro^71^. For the MGI phenotype ontology, we downloaded the MGI table MGI_PhenoGenoMP.rpt, which lists knockout phenotypes, and used the MGI table MGI_Gene_Model_Coord.rpt to convert mouse MGI gene identifiers to mouse Ensembl gene identifiers. We used 1:1 orthologous coding genes downloaded from Ensembl BioMart^64,72^ to map mouse to human Ensembl gene identifiers.

### Forward genomics

To identify genes that are preferentially lost in independent lineages that share a particular phenotypic change, we developed a new variant of the forward genomics method. While the original forward genomics approach searches for genomic regions that have a higher nucleotide divergence in species that share an independently evolved phenotype^6,9^, the new method utilizes the maximum percentage of the intact reading frame (%intact, described above) to search for genes that are lost preferentially in these species. Then, we used the phylogenetic generalized least squares (pGLS) approach^9,45^ to search for genes that have a lower %intact value (indicating gene loss) preferentially in species with the derived phenotype. This approach takes the phylogenetic relatedness of the species as given by the phylogenetic tree into account.

We applied this new approach to three phenotypes: (i) the loss of tooth enamel, (ii) the presence of scales, and (iii) a fully aquatic lifestyle (Supplementary Tables 6–8). Species with the derived phenotype (teeth/enamel-less, scales, etc.) where assigned to trait-group 1 while the remaining species were assigned to trait-group 2. We excluded genes that had missing data due to assembly gaps for more than 50% of the trait-group 1 or trait-group 2 species. Since we aimed at detecting genes that are not only lost in trait-group 1, but are also preferentially intact in the trait-group 2 species, we further excluded genes where less than 90% of the trait-group 2 species had %intact ≥90% or where ≥5% of the trait-group 2 species had %intact <60%. Genes were then ranked by the *p* value based on the slope of the pGLS regression fit and genes with a *p* value <10^−6^ were selected.

### Dating gene loss events

For all genes that did not exhibit inactivating mutations that are shared between related gene loss species, we used the procedure described in refs. ^13,73^. Given a branch along which the gene was lost, this approach makes the assumption that a gene evolved under a selective pressure similar to that in other species until it was inactivated. After the loss, the gene is assumed to evolve neutrally and should accumulate synonymous and non-synonymous mutations at an equal rate. The *K*_a_/*K*_s_ value (*K*) estimated for this entire branch is then the average of the *K*_a_/*K*_s_ value for the part of the branch, where the gene was under selection (*K*_s_) and the *K*_a_/*K*_s_ value for the part of the branch, where the gene evolved neutrally (*K*_n_ = 1), weighted by the proportion of time for which the gene was evolving under selection (*T*_s_/*T*) and neutrally (*T*_n_/*T*):

$$K = K_{\rm{s}} \ast T_{\rm{s}}/T + K_{\rm{n}} \ast T_{\rm{n}}/T,$$

where *T* represents the time since the split from the last common ancestor. We used the *K*_a_/*K*_s_ value for mammals with a functional gene (*K*_s_) and used the lower and upper bound of the confidence interval for the species divergence time *T* from TimeTree^74^ to estimate a lower and upper bound for *T*_*n*_ as

$$T_{\rm{n}} = T \ast \left( {K - K_{\rm{s}}} \right)/\left( {1 - K_{\rm{s}}} \right),$$

which provides an estimate of how long the gene has been evolving neutrally.

### Code availability

All custom scripts are available on request from the corresponding author.

### Data availability

The data sets generated during the current study are available from the corresponding author on reasonable request.

## Electronic supplementary material

Supplementary Information(PDF 1895 kb)

Description of Additional Supplementary Files(PDF 167 kb)

Supplementary Data 1(XLSX 193 kb)
