# Author Correction: A genomics approach reveals insights into the importance of gene losses for mammalian adaptations

**DOI:** 10.1038/s41467-019-13828-5

**Published:** 2019-12-10

**Authors:** Virag Sharma, Nikolai Hecker, Juliana G. Roscito, Leo Foerster, Bjoern E. Langer, Michael Hiller

**Affiliations:** 1 Max Planck Institute of Molecular Cell Biology and Genetics, Pfotenhauerstr. 108, 01307 Dresden, Germany; 2 Max Planck Institute for the Physics of Complex Systems, Noethnitzer Str. 38, 01187 Dresden, Germany; 3 Center for Systems Biology Dresden, Pfotenhauerstr. 108, 01307 Dresden, Germany

**Keywords:** Genome informatics, Evolutionary genetics, Molecular evolution

Correction to: *Nature Communications* 10.1038/s41467-018-03667-1, published online 23 March 2018.

The original version of this Article contained an error in the email address of the corresponding author. This has now been corrected in both the PDF and HTML versions of the Article.

