# Supplementary material for: A genomics approach reveals insights into the importance of gene losses for mammalian adaptations
Source: Nat Commun. 2018 Mar 23;9:1215. doi: 10.1038/s41467-018-03667-1 (PMC5865188; doi:10.1038/s41467-018-03667-1)
Supplement: Supplementary file 1 — Supplementary Information(PDF 1895 kb) [file 41467_2018_3667_MOESM1_ESM.pdf]

Supplementary Information

A genomics approach reveals insights into the importance of gene losses for mammalian adaptations

Sharma *et al.*

The Supplementary Information contains

- Supplementary Figures 1 - 35
- Supplementary Tables 1 - 8
- Supplementary Notes 1 - 8

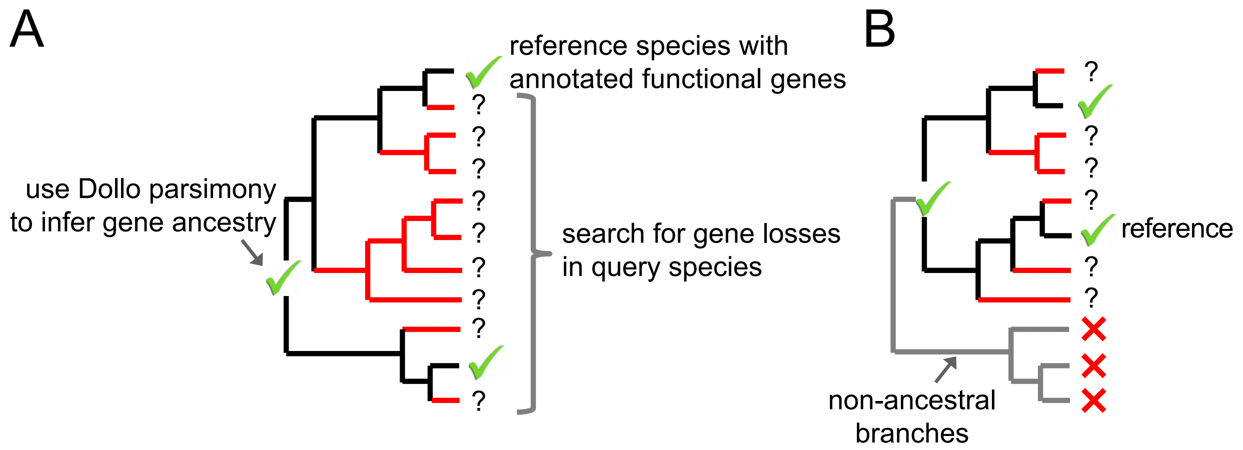

Supplementary Figure 1: General framework for detecting gene losses in genome alignments.

(A) Our approach considers all coding genes that are annotated and thus likely functional in a chosen reference species. We detect loss of a given gene in other query species by searching genome alignments for gene-inactivating mutations. Genome alignments are well-suited to detect gene losses for the following reasons. First, genome alignments can reveal the remnants of inactivated but not completely deleted genes, even if these genes are not expressed anymore and thus are not contained in a transcriptome or in mRNA/protein databases. Second, splice site mutations, which are one important class of inactivating mutations, can only be detected at the genomic but not at the mRNA/protein level. Third, information about missing sequence (assembly gaps, regions of low sequencing quality) are only visible by direct genome analysis. This is important as the absence of a gene in a gene/protein database or in a genomic BLAST run cannot distinguish between artifacts that perfectly mimic absence of a gene (such as large assembly gaps) and the complete deletion of a gene. Since gene loss in a query species requires that the common ancestor of the reference and this query species possessed the gene, we used Dollo parsimony to infer gene ancestry based on query species where the gene lacks any gene-inactivating mutations. In the illustrated case, the gene was likely present in the common ancestor of all species, and thus could be lost along any of the red branches in query species that descend from that ancestor.

(B) To detect gene loss events in the species that was chosen as the reference in (A), the approach can be repeated by selecting a different reference species. This example also illustrates that the presence of inactivating mutations (or no aligning sequence) in the 3 most basal species will not be considered as gene loss in these species since they do not descend from an ancestor that possessed the gene.

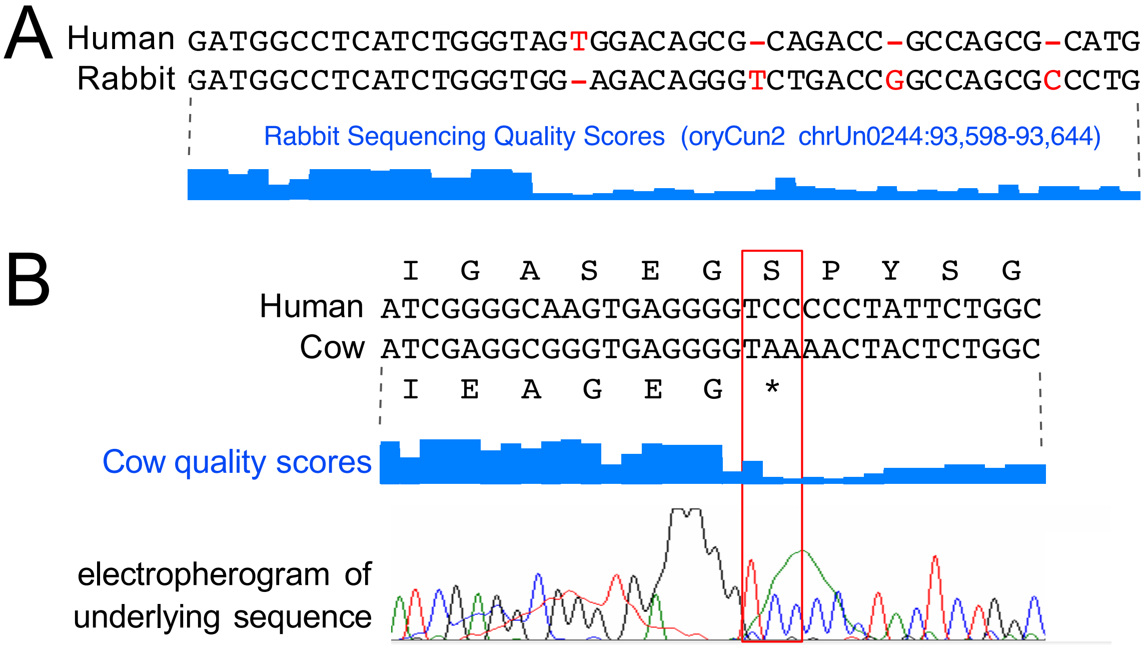

Supplementary Figure 2: Sequencing errors mimic gene-inactivating mutations.
(A) The alignment of the third exon of the human *ARL2* gene reveals several frameshifting insertions and deletions in the rabbit. These gene-inactivating mutations are likely sequencing errors as the corresponding bases in rabbit have very low sequencing quality scores.
(B) The last exon of human *ARHGAP33* reveals an in-frame stop codon in the alignment to the cow 2007 genome assembly (bosTau4). As shown by the quality score track and the electropherogram, the stop codon mutation is in fact a sequencing error that was fixed in later assemblies of the cow genome.
Our approach made use of sequence quality scores, where available, to replace all genomic bases of poor quality (Phred score <40) by an "N" character, which were subsequently ignored in the search for inactivating mutations.

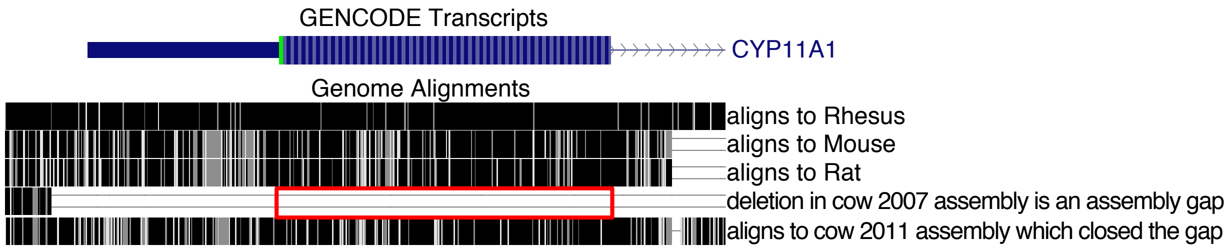

Supplementary Figure 3: Assembly gaps mimic exon or gene deletions.
Genome assembly gaps indicate regions where parts of the real genome are missing in the given assembly. Missing sequence can comprise exons or even entire genes and, consequently, can mimic larger deletions of exons or genes, which would otherwise be indicative of gene loss. In this example, the first exon (blue box) of the human *CYP11A1* gene aligns to rhesus, mouse, rat and many other mammals (black parts visualize aligning sequence); however, this exon appears to be deleted in the cow 2007 assembly (bosTau4, double horizontal lines), where it overlaps an assembly gap. Indeed, the 2011 bosTau7 assembly resolves this assembly gap and shows that this exon actually aligns to the cow.

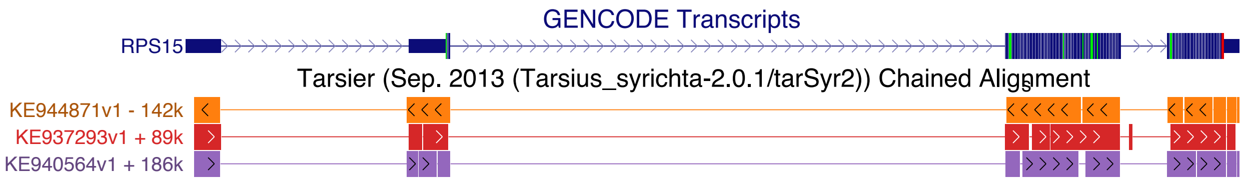

Supplementary Figure 4: Alignments between a gene and a processed pseudogene copy or a paralog may lead to the incorrect inference of gene loss.
The ortholog of the human *RPS15* gene (blue boxes are exons, blue lines are introns) is not present in the genome assembly of the tarsier, however several processed *RPS15* pseudogenes align instead (boxes in an "alignment chain" represent aligning regions, the single horizontal lines show the lack of all introns, which is a hallmark of a processed pseudogene). In contrast to orthologs, both paralogs and pseudogenes are often located in a different context, resulting in aligning chains that span only a single gene, as shown here. Since processed pseudogenes often evolve neutrally, they can accumulate inactivating mutations, which would then incorrectly be taken as evidence that *RPS15* is lost in the tarsier.

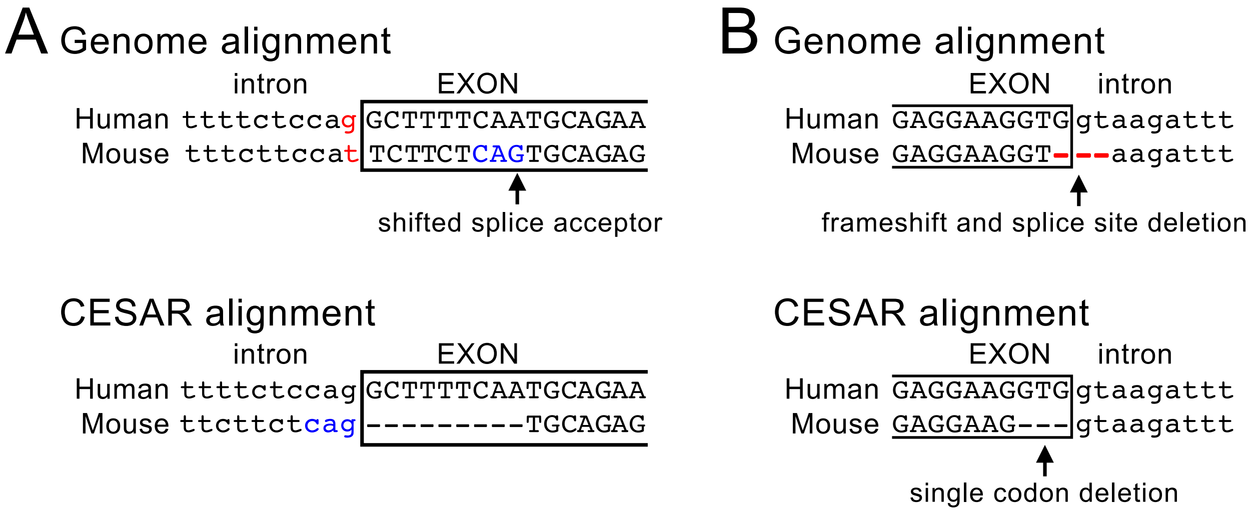

Supplementary Figure 5: Evolutionary splice site shifts and alignment ambiguities mimic gene-inactivating mutations.

(A) The genome alignment shows that the acceptor of exon 5 of human *C1orf168* is mutated in mouse, which inactivates this splice site. However, the mouse acceptor site CAG (highlighted in blue) is shifted by 9 bases into the exon, making the exon three codons shorter. CESAR [1] aligns the shifted mouse acceptor splice site to the human acceptor splice site, and thus recognizes that this exon in mouse has a consensus splice site.

(B) Genome alignment tools are not aware of the protein's reading frame and the position of splice sites but instead align nucleotide sequences without any annotation. Here, the genome alignment shows a frameshifting 1 bp deletion and the deletion of the donor splice site in mouse at the end of exon 11 of the human *ICA1* gene. This is an alignment ambiguity since CESAR reports an alternative alignment where the three bp deletion is shifted such that a single codon is deleted and an intact splice site is present.

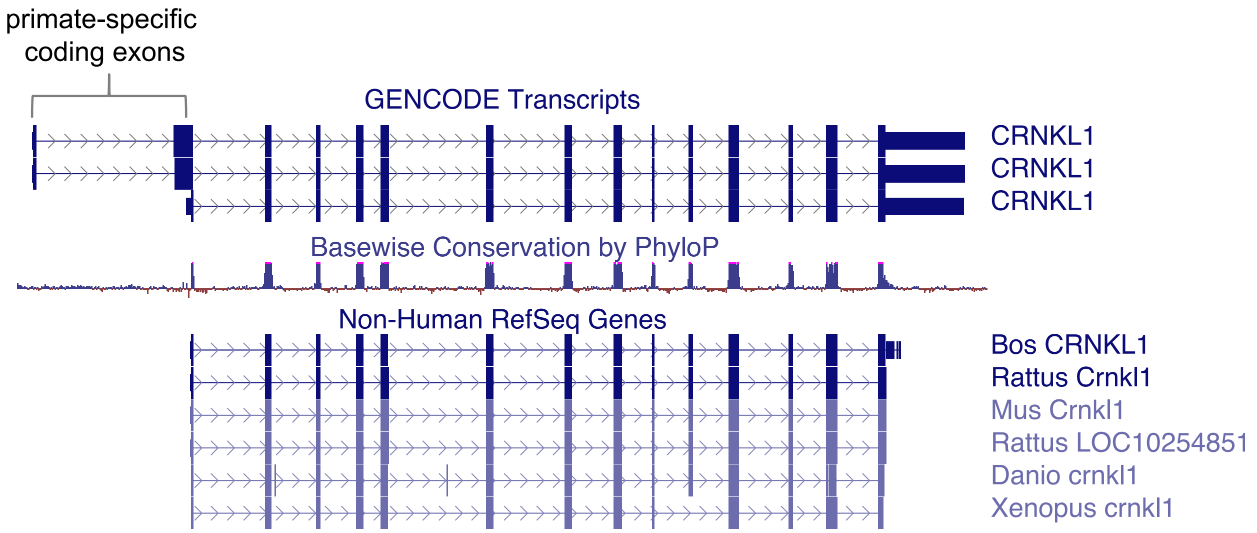

Supplementary Figure 6: Transcripts that contain non-ancestral exons can incorrectly indicate gene loss.

It is common practice to use the transcript with the longest reading frame when selecting a representative transcript of a gene, which makes the assumption that coding exons are typically well conserved. However, as shown here, this assumption is not always true: the first two coding exons of the longest transcript of *CRNKL1* are primate-specific and do not show sequence conservation (PhyloP track) in vertebrates. These exons exhibit gene-inactivating mutations in non-primate species, which could incorrectly indicate the loss of this gene. In contrast, all exons of the shorter transcript are ancestral as they occur in cow, rat, mouse and frog ("Non-human RefSeq Genes") and no inactivating mutation is detected in the exons of the shorter transcript.

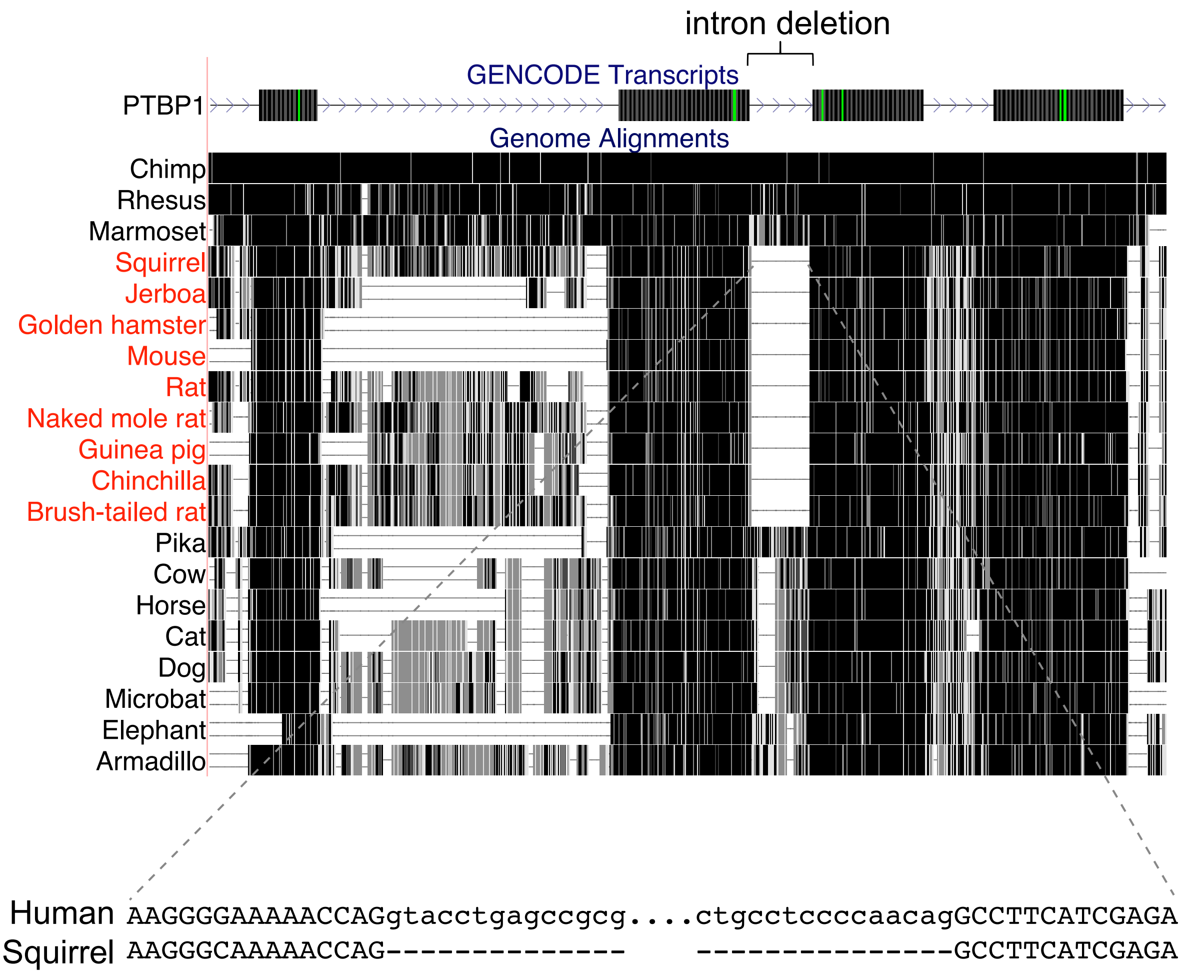

Supplementary Figure 7: Precise intron deletions mimic splice site mutations.
An intron of the *PTBP1* gene is deleted in the entire rodent clade (red font), as shown by the single line in the genome alignment visualization. This deletion precisely removes the intron, as shown by the sequence alignment between human and squirrel (exonic bases are in upper case, intron bases are in lower case). While the deletion of splice sites is indicative of gene loss, the precise deletion of an entire intron, as shown here, should not be taken as evidence for gene loss as it simply results in a larger composite exon in the query species. To detect such cases automatically, we ran CESAR on a reference sequence consisting of both exons without the intron. If CESAR reported an intact reading frame for the composite exon in the query, we did not consider the splice site deletions as inactivating mutations.

A human-mouse alignment with two compensating frameshifts hg38: chr15:40,340,838-40,340,923

```
R  Q  G  E  A  G  R  G  P  A  P  V  A  P  A  F  L  P  L  W  L  P  R  G  C  S  G  I  L
GGCAGGGAGAGGCCGGCAGAGGCCCGGCTCCGGTGGCTCCAGCTTTCCTCCCACTCTGGCTCCCAGGGGCTGCTCTGGAATTCTC
||||||||||||||| ||| |  ||||||| |||||||||||||| |||||  |||||||| ||| || |||| | || |||||||
GGCAGGGAGAGGCCGACAGGG--CCGGCTCTGGTGGCTCCAGCTTCCCTCCAGCTCTGGCT-CCCGGGAGCTGTTTTGAAATTCTC
R  Q  G  E  A  D  R     A  G  S  G  G  S  S  F  P  P  A  L  A     P  G  S  C  F  E  I  L
```

B alignment without frameshifting indels

```
GGCAGGGAGAGGCCGGCAGAGGCCCGGCTCCGGTGGCTCCAGCTTTCCTCCCACTCTGGCTCCCAGGGGCTGCTCTGGAATTCTC
||||||||||||||| ||| | |    ||      |   |    ||||  |||     |   ||| || |||| | || |||||||
GGCAGGGAGAGGCCGACAGGGCCGGCTCTGGT---GGCTCCAGCTTCCCTCCAGCTCTGGCTCCCGGGAGCTGTTTTGAAATTCTC
```

Supplementary Figure 8: Compensating frameshifting insertions and deletions.
(A) A frameshifting indel results in a reading frame shift; however, downstream frameshifting indels can compensate for the first frameshift by restoring the ancestral reading frame. In this example, the alignment between the human *C15orf52* gene and its mouse ortholog shows a 2 bp frameshifting deletion followed by another deletion of 1 bp (red font), which restores the ancestral reading frame. Since compensating frameshift mutations do not provide conclusive evidence for gene loss, our pipeline scanned for and excluded such compensatory frameshifts that (i) restore the ancestral reading frame and (ii) where the frame-shifted sequence (blue font) is translatable into the new reading frame without encountering in-frame stop codons.
(B) An alternative alignment that avoids the two frameshifts has a substantially lower sequence similarity. Thus, the alignment in (A) is strongly favored, indicating that both compensating frameshifting mutations actually occurred during evolution.

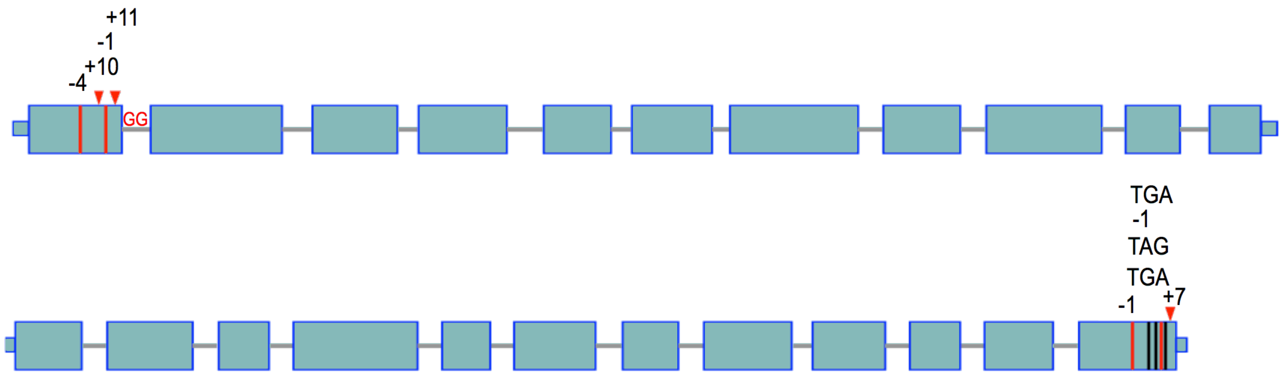

Supplementary Figure 9: Inactivating mutations that occur at the beginning or end of a gene do not indicate gene loss.

The figure shows the exon-intron structure of two genes where inactivating mutations solely occur close to the N- or C-terminus of the encoded protein. Top: The *P4HB* gene in elephant exhibits only inactivating mutations in the first exon and probably uses a downstream start codon. Bottom: The *MTO1* gene in mouse has inactivating mutations in the last exon, which changes the C-terminus of the encoded protein.

Studies of gene-inactivating mutations in the human population [2] and across mammals [1] consistently showed that protein termini are under less evolutionary constraint, likely because extensions or truncations are less likely to affect function. For this reason, we do not consider inactivating mutations within the first or last 20% of the protein as evidence for gene loss.

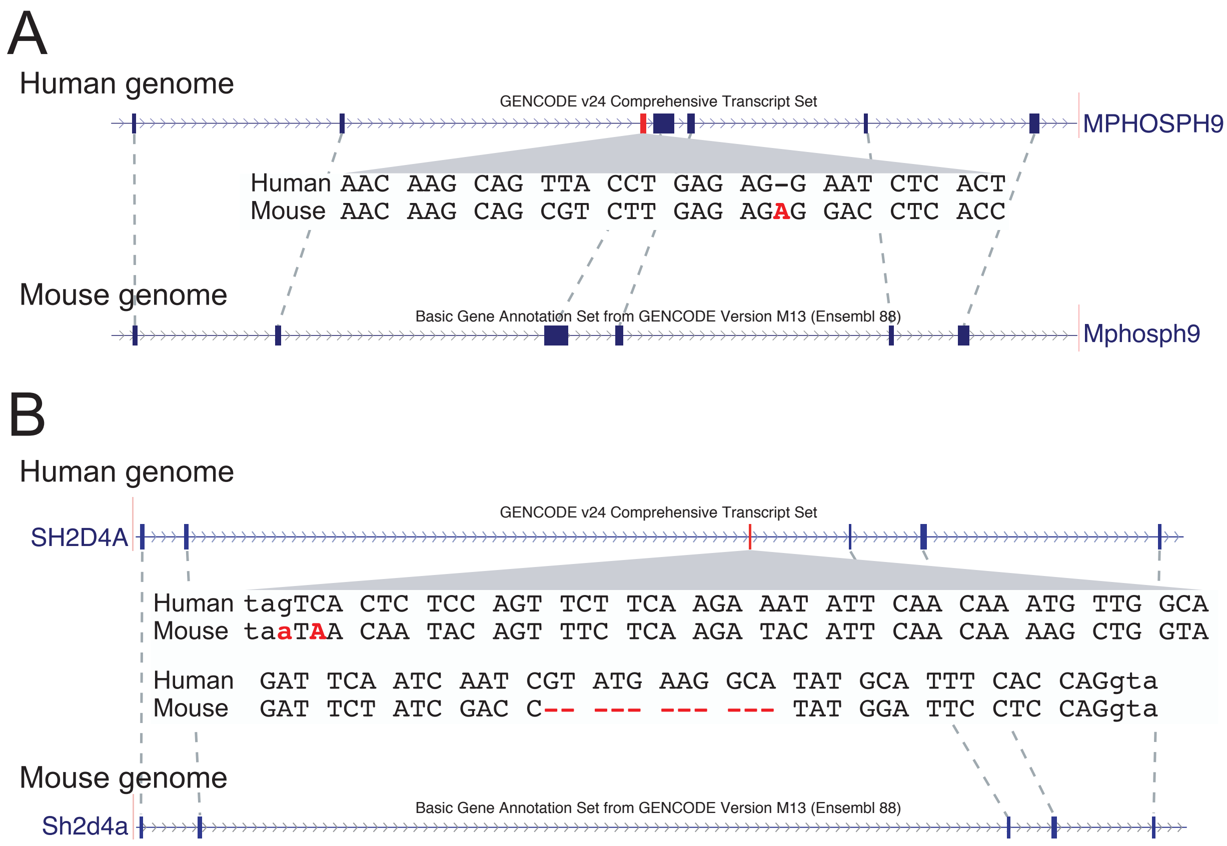

Supplementary Figure 10: Lost exons in conserved genes.

(A) The comparison of the exon-intron structures of the annotated human and mouse *MPHOSPH9* gene shows that mouse has lost an exon (red box) in the otherwise conserved gene. This exon has a frameshifting 1 bp insertion (red).

(B) The conserved *SH2D4A* gene also lost an exon in mouse (red box). This exon has a mutation that destroys the acceptor splice site (AG → AA), a stop codon mutation and an 11 bp frameshifting deletion.

These examples show that gene-inactivating mutations in a single exon does not provide sufficient evidence for the loss of the entire gene.

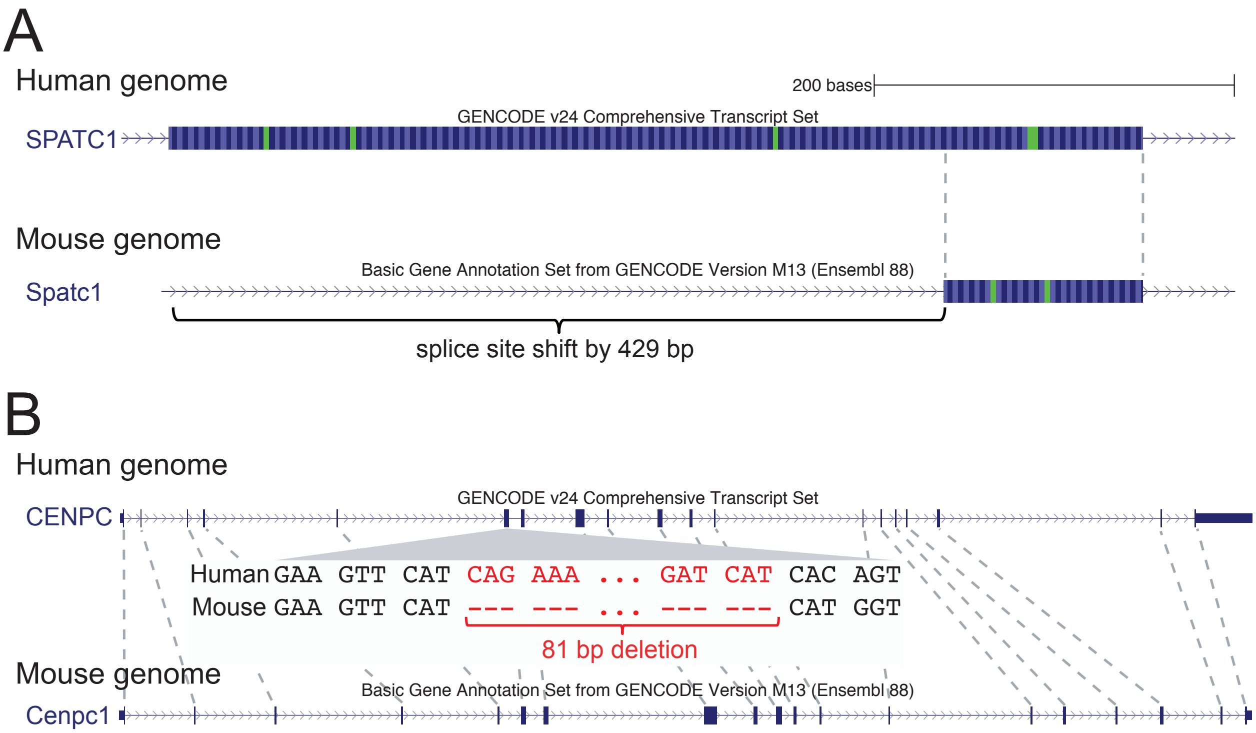

Supplementary Figure 11: Exons that are not entirely conserved in otherwise conserved genes.

(A) Large splice site shift in the *SPATC1* gene: While exon 3 of the human gene is 540 bp long, the mouse exon is only 111 bp long due to a 429 bp shift of the acceptor site. CESAR is not able to recognize splice site shifts over such large distances. However, the substantial exon size change results in the loss of 143 amino acids in this 591-amino acid protein, which indicates that the function of the gene is not fully conserved.

(B) Large deletion in a conserved exon of *CENPC* gene: While human exon 6 is 286 bp long, the mouse exon is 202 bp long due to an 81 bp and another 3 bp deletion.

Together with the lost individual exons shown in Supplementary Figure 10, these examples of large exon size changes due to frame-preserving deletions or splice site shifts show that mutations in a single exon are not sufficient to infer loss of the entire gene.

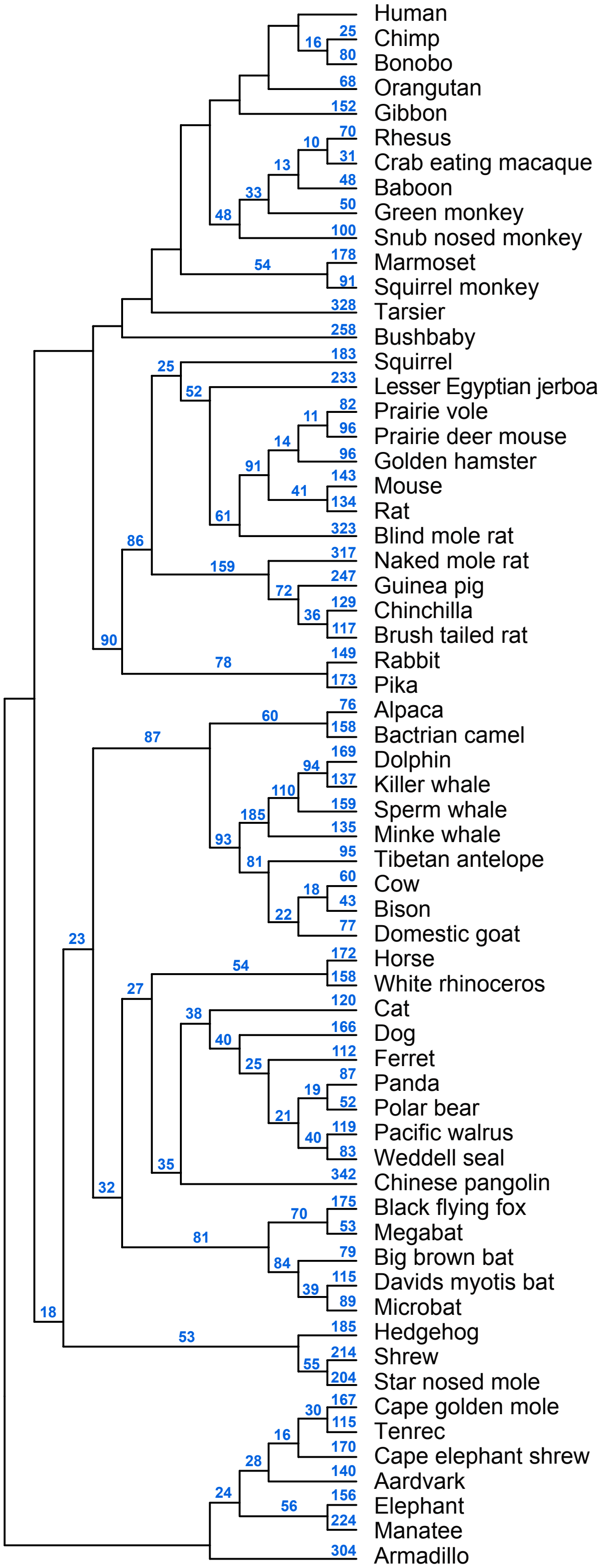

Supplementary Figure 12: Overview of all detected gene loss events.
We used Dollo parsimony to assign gene losses to the branches in the phylogenetic tree.
The number is shown in blue font.

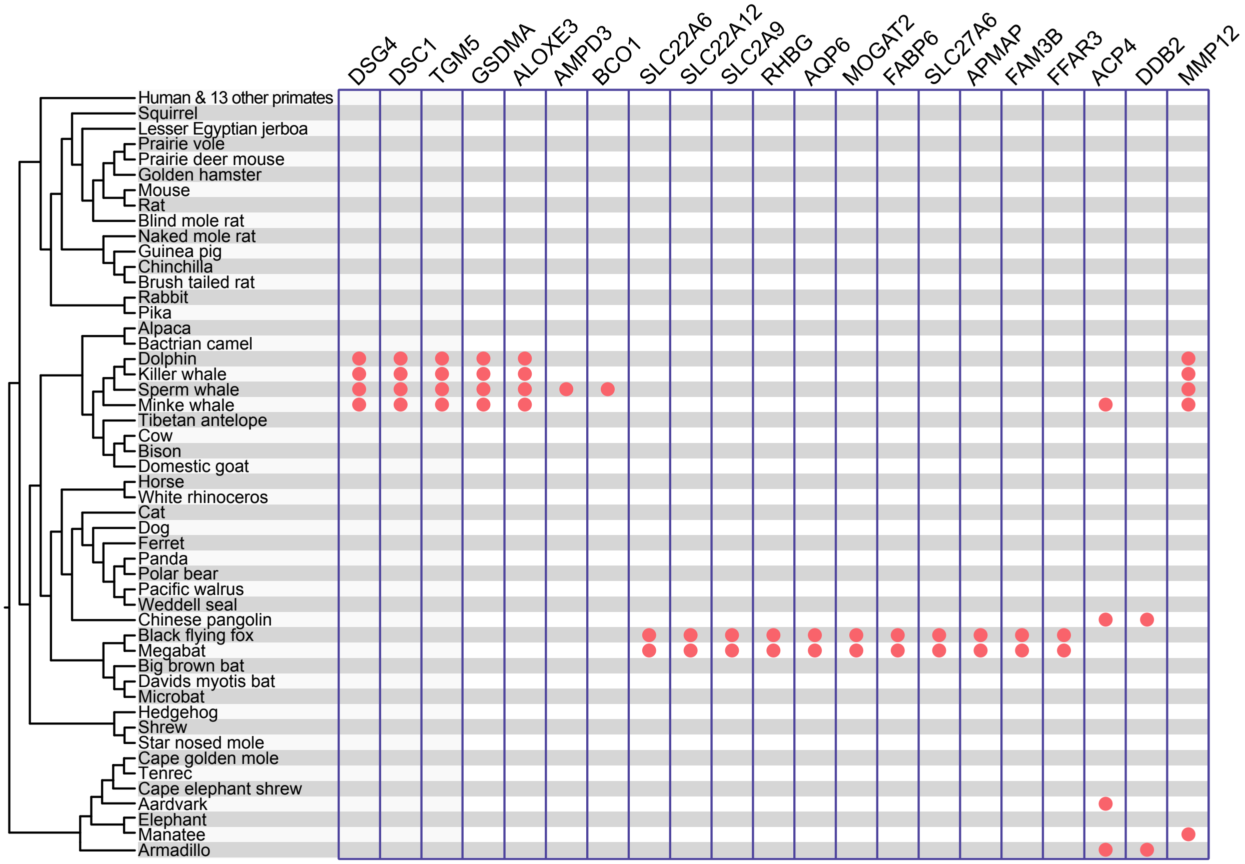

Supplementary Figure 13: Previously unknown gene losses that are discussed in the manuscript.

A red dot indicates the species that have lost these genes. The three genes shown in Figure 5 (*ACP4*, *DDB2* and *MMP12*) are repeated here for completeness.

.

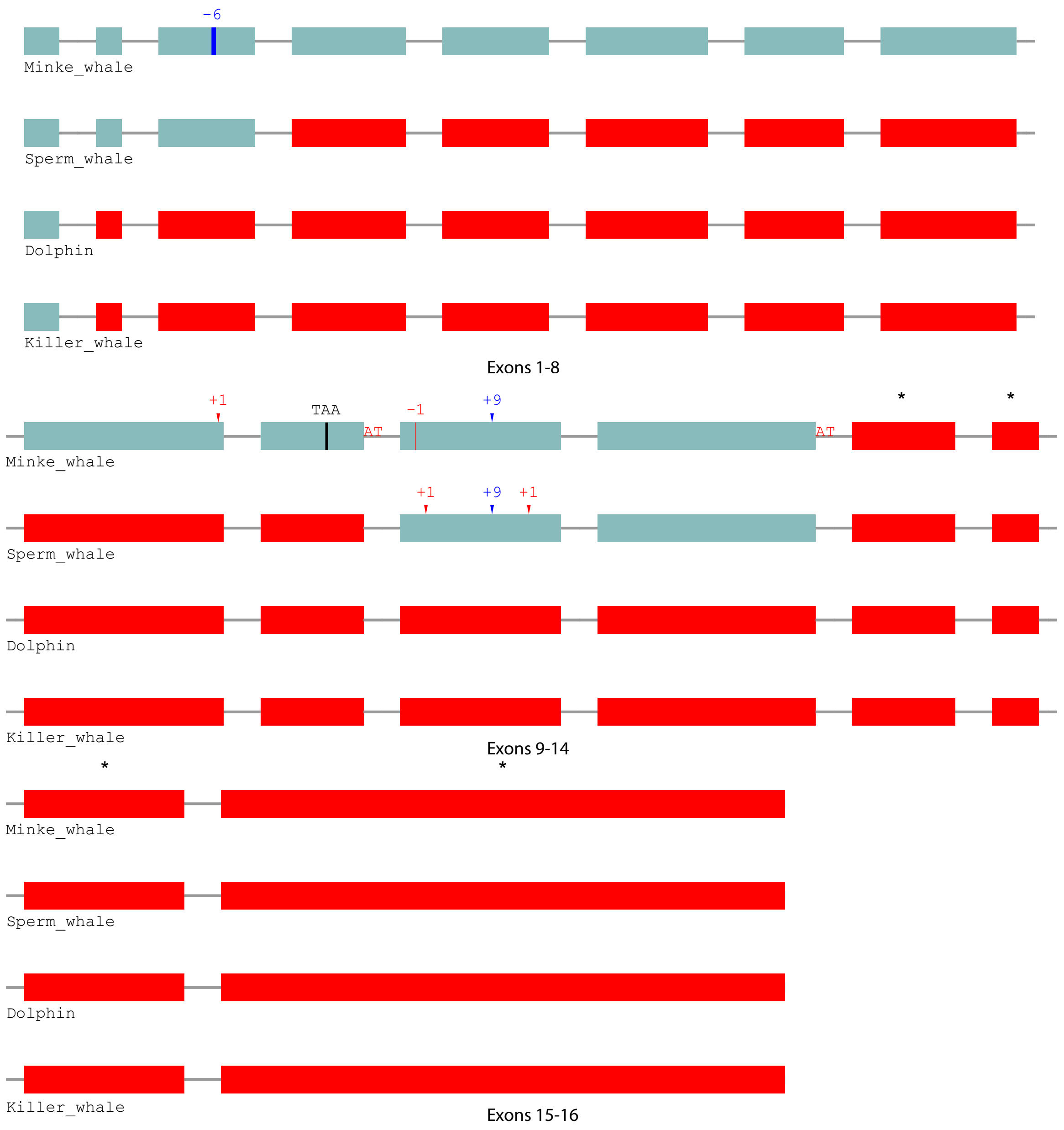

Supplementary Figure 14: Mutations in *DSG4* in cetaceans.
Visualization: Boxes are exons (proportional to their size), introns are horizontal lines. A filled red box is an exon deletion, a filled grey box indicates missing sequence due to assembly gaps, a black vertical line is an in-frame stop codon, a red (blue) vertical line is a frameshifting (frame-preserving) deletion, a red (blue) triangle is a frameshifting (frame-preserving) insertion. Splice site mutations are indicated before or after the exon.
Inspecting the alignment chains reveals that the deletion of exons 13-16 (highlighted by asterisks) shares the same breakpoint in the minke and sperm whale, showing that this deletion and thus *DSG4* loss already happened in the cetacean ancestor before the split of the toothed and baleen whale lineage.

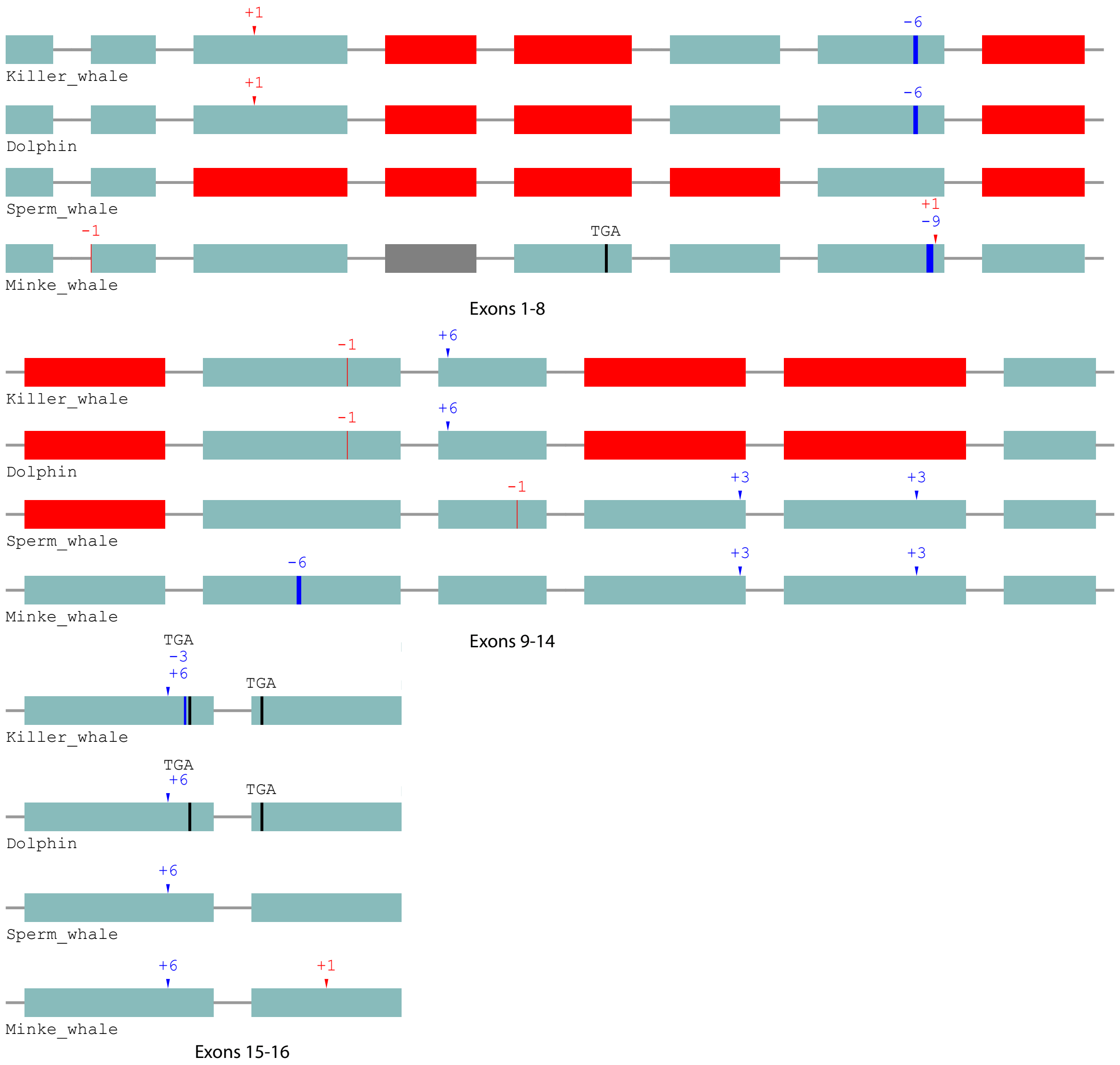

Supplementary Figure 15: Mutations in *DSC1* in cetaceans.

While there are two deletions covering exons 4-5 and 8-9 that are shared between the three toothed whales (evident from shared deletion breakpoints), there is no obvious gene-inactivating mutation that is shared between all four cetaceans. Therefore, we estimated when the loss of *DSC1* happened. Ka/Ks ratios of ~1 for the branch leading to minke whale and 0.89 for the branch leading to the toothed whale ancestor (Supplementary Table 5) suggest that the loss of *DSC1* overlaps the split of the cetacean ancestor or happened soon after. This indicates that *DSG4* (the specific binding partner of *DSC1*) was lost first, followed by the subsequent loss of *DSC1*. However, we cannot exclude the possibility that another mutation (different from frameshifts, stop codon and splice site mutations) lead to loss of *DSC1* function before the loss of *DSG4*. In either case, the loss of the upper epidermis desmosome components *DSG4* and *DSC1* coincided with the period during which epidermal adaptations evolved in cetaceans.

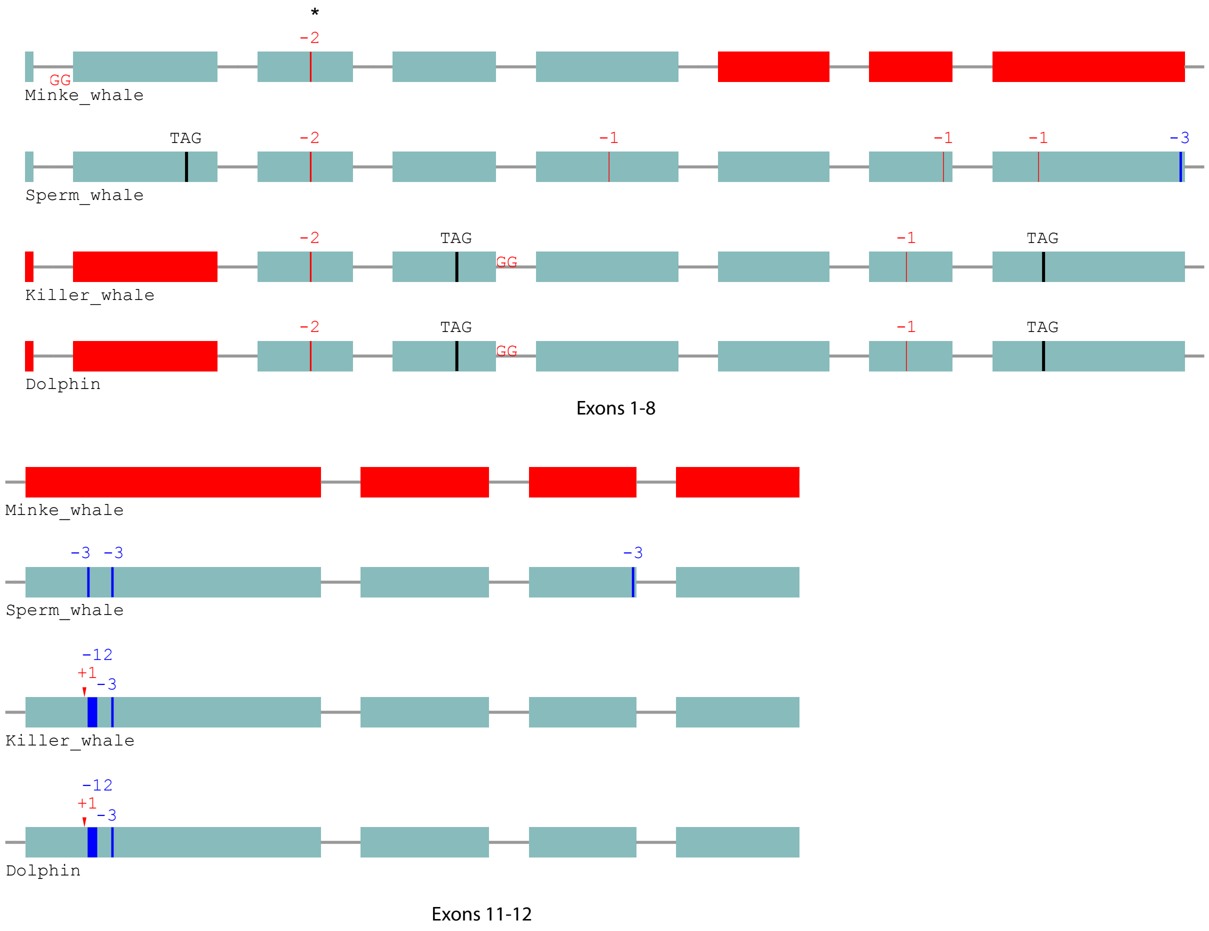

Supplementary Figure 16: Mutations in *TGM5* in cetaceans.

This gene shares a frameshifting 2 bp deletion in exon 2 in all four species (asterisk), showing that *TGM5* was already lost before the split of the toothed and baleen whale lineage.

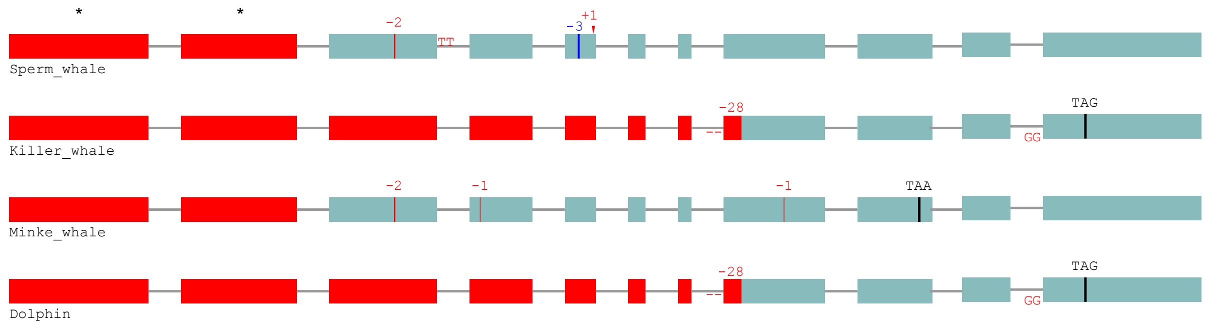

Supplementary Figure 17: Mutations in *GSDMA* in cetaceans.
The joint deletion of the promoter (not shown in the figure) and exons 1 and 2 (asterisks) shares the same breakpoint in all four species as revealed by the alignment chains, showing that this deletion and thus *GSMDA* loss already happened in the cetacean ancestor before the split of the toothed and baleen whale lineage.

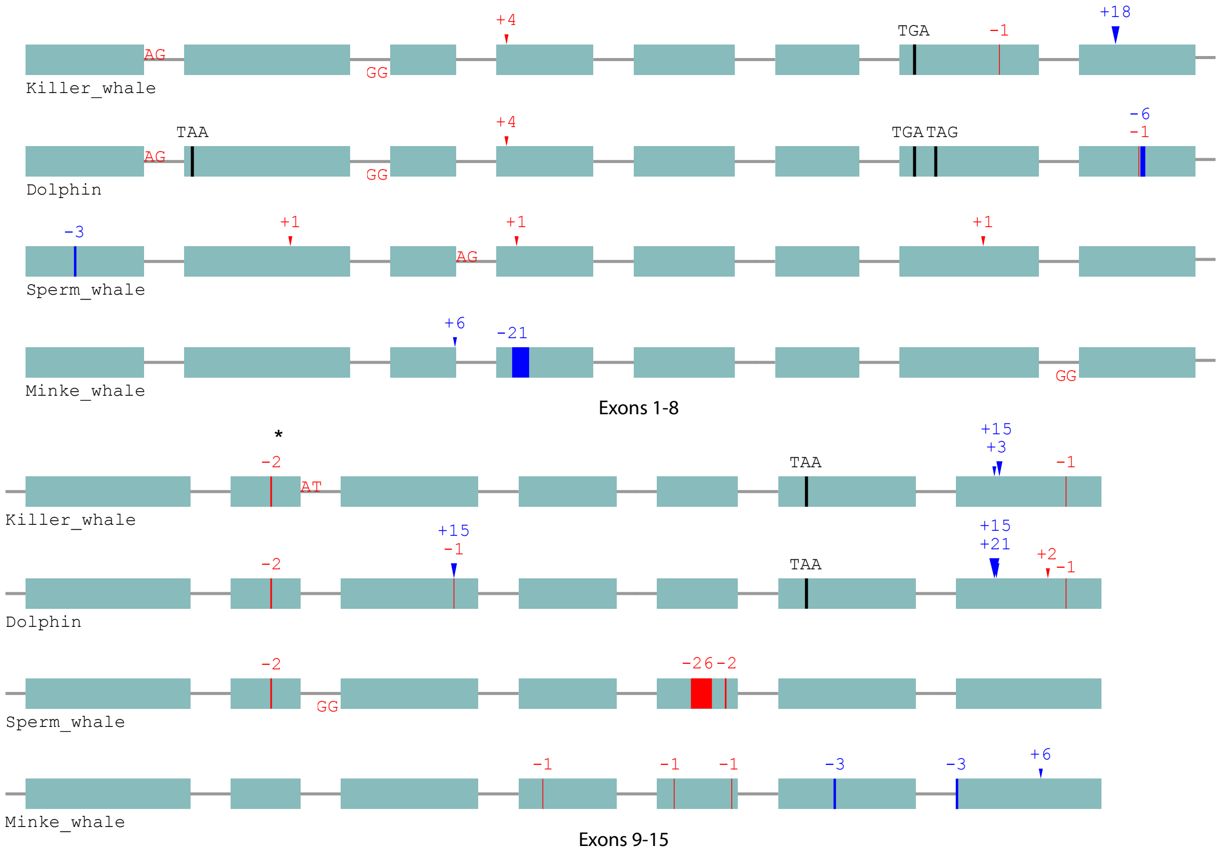

Supplementary Figure 18: Mutations in *ALOXE3* in cetaceans.
While a 2 bp deletion (asterisk) shows that this gene was already lost in the toothed whale ancestor, there is no obvious gene-inactivating mutation that is shared between the toothed and baleen whale lineage. Consistent with an independent gene loss after the split of the cetacean ancestor, we estimated that *ALOXE3* was lost in the last 10 My along the branch leading to the minke whale (Supplementary Table 5).

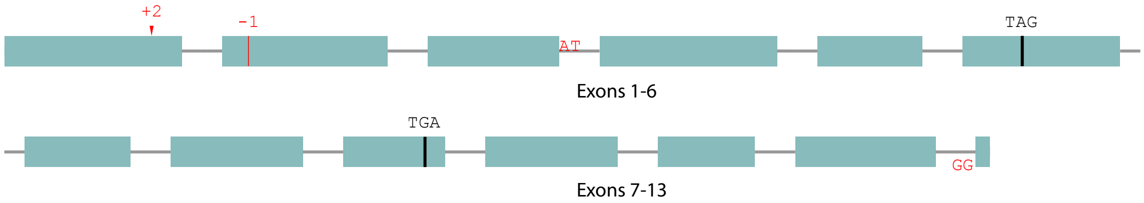

Supplementary Figure 19: Mutations in *AMPD3* in sperm whale.
Since sperm whale is the only species in our dataset that lost *AMPD3*, we determined the Ka/Ks values for this gene, which suggests that *AMPD3* was evolving neutrally for all or most of the terminal branch leading to the sperm whale (Supplementary Table 5). This suggests that gene loss happened soon after the sperm whale lineage split from the other toothed whales 31-37 Mya (www.timetree.org). This estimate also indicates that the loss predates the split of Physeter and its long and deep diving sister species (pygmy and dwarf sperm whale) 23.2-29.5 Mya (www.timetree.org).

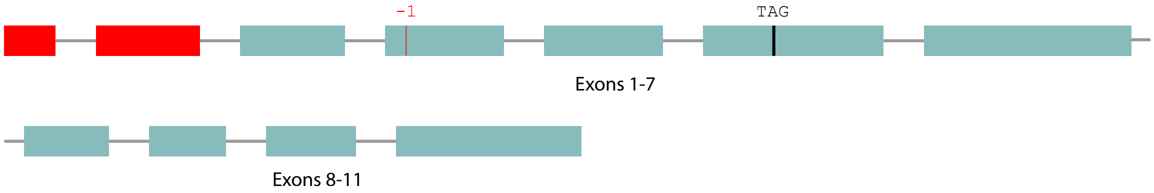

Supplementary Figure 20: Mutations in *BCO1* in sperm whale.
Since sperm whale is the only species in our dataset that lost *BCO1*, we used the method described in references [3,4] to date the gene loss, which indicates that the loss of *BCO1* happened soon after the sperm whale lineage split from the other toothed whales 31-37 Mya (www.timetree.org, Supplementary Table 5).

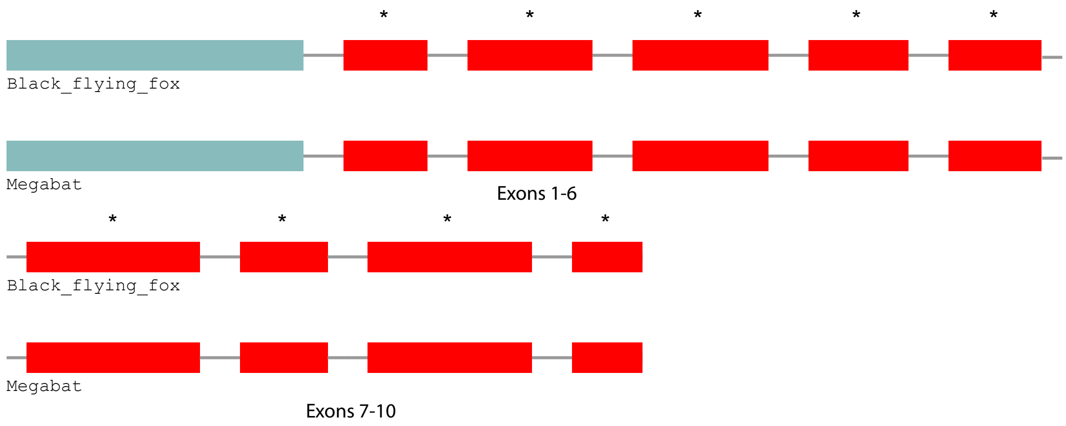

Supplementary Figure 21: Mutations in *SLC22A6* in the black flying fox and the large flying fox (megabat).
Since the large deletion (asterisks) shares the same breakpoints (evident from the alignment chains) in both fruit bats, the loss of *SLC22A6* already happened in the ancestor of both species.

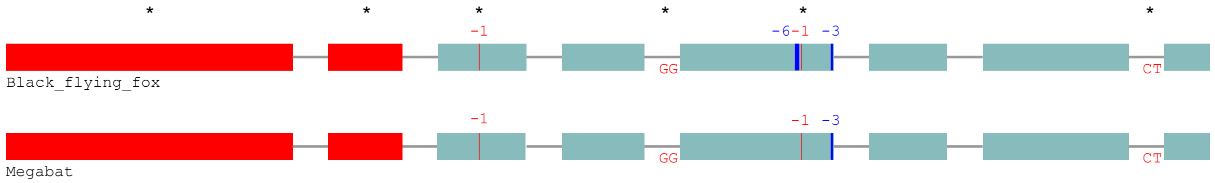

Supplementary Figure 22: Mutations in *SLC22A12* in the black flying fox and the large flying fox.
Apart from a shared deletion of the first two exons, several other shared inactivating mutations (asterisks) show that *SLC22A12* loss already happened in the ancestor of both fruit bats.

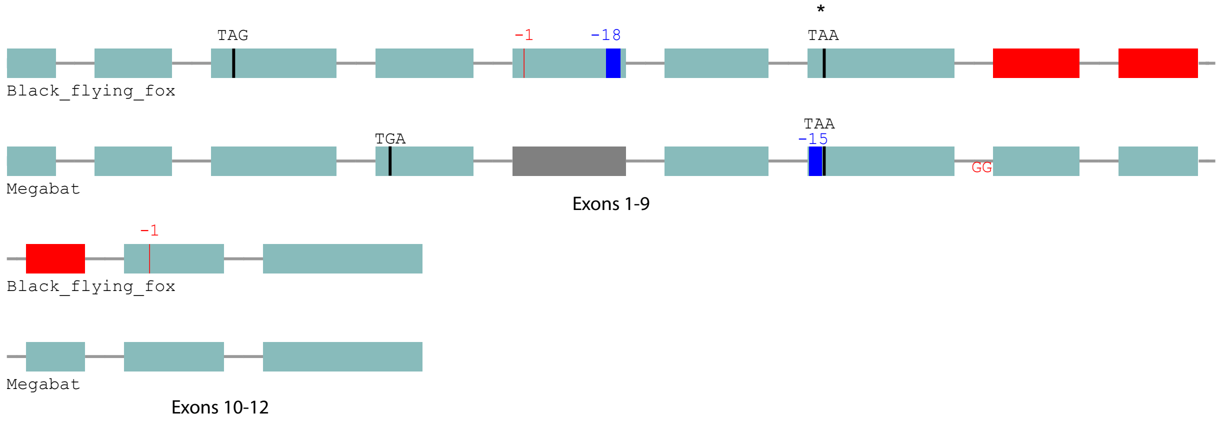

Supplementary Figure 23: Mutations in *SLC2A9* in the black flying fox and the large flying fox.
A shared stop codon in exon 7 (asterisk) shows that the loss of *SLC22A9* already happened in the ancestor of both fruit bats.

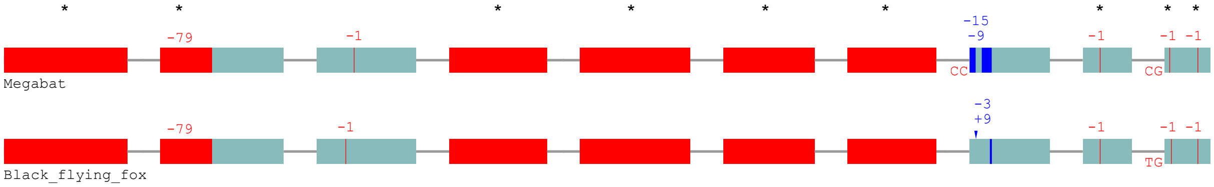

Supplementary Figure 24: Mutations in *RHBG* in the black flying fox and the large flying fox.
Several shared inactivating mutations (asterisks) show that the loss of *RHBG* already happened in the ancestor of both fruit bats.

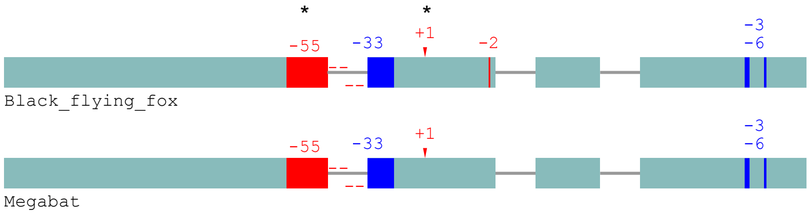

Supplementary Figure 25: Mutations in *AQP6* in the black flying fox and the large flying fox.
Shared inactivating mutations (asterisks) show that *AQP6* was already lost the common ancestor of both fruit bats.

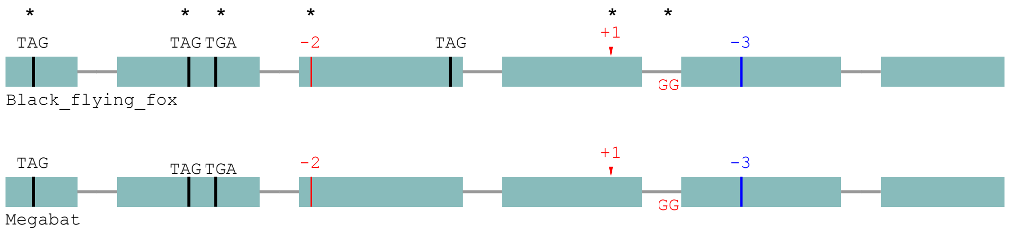

Supplementary Figure 26: Mutations in *MOGAT2* in the black flying fox and the large flying fox.

Several shared inactivating mutations (asterisks) show that the loss of *MOGAT2* already happened in the ancestor of both fruit bats.

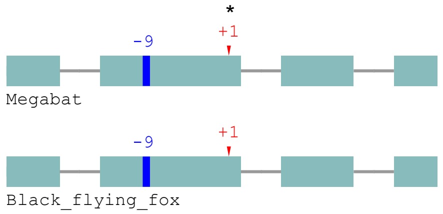

Supplementary Figure 27: Mutation in *FABP6* in the black flying fox and the large flying fox.

The shared frameshift in exon 2 (asterisk) shows that the loss of *FABP6* already happened in the ancestor of both fruit bats.

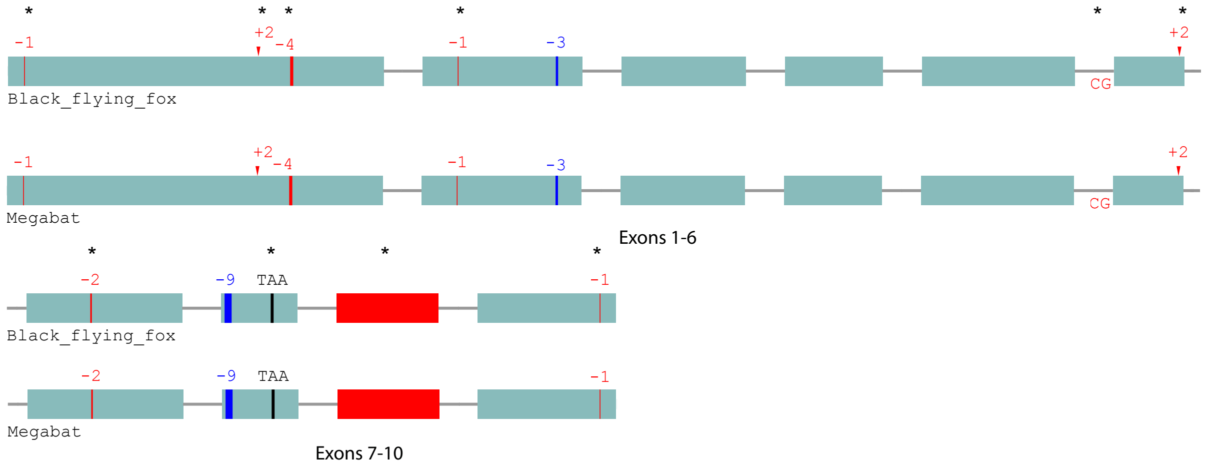

Supplementary Figure 28: Mutations in *SLC27A6* in the black flying fox and the large flying fox.

Several shared inactivating mutations (asterisks) show that the loss of *SLC27A6* already happened in the ancestor of both fruit bats.

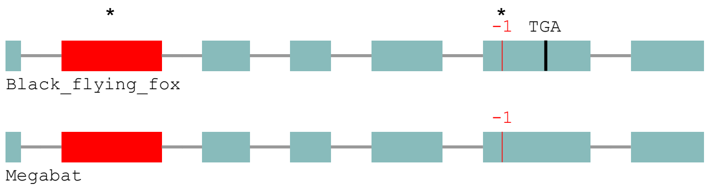

Supplementary Figure 29: Mutations in *FAM3B*

A shared frameshifting deletion in exon 6 and the deletion of exon 2 with the same breakpoints (asterisks) show that the loss of *FAM3B* already happened in the ancestor of both fruit bats.

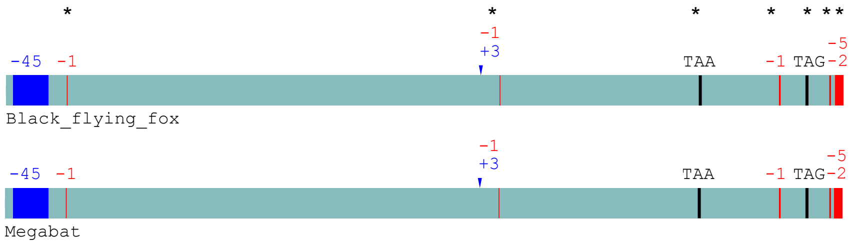

Supplementary Figure 30: Mutations in *FFAR3*

Several shared inactivating mutations (asterisks) show that *FFAR3* was already lost in the ancestor of both fruit bats.

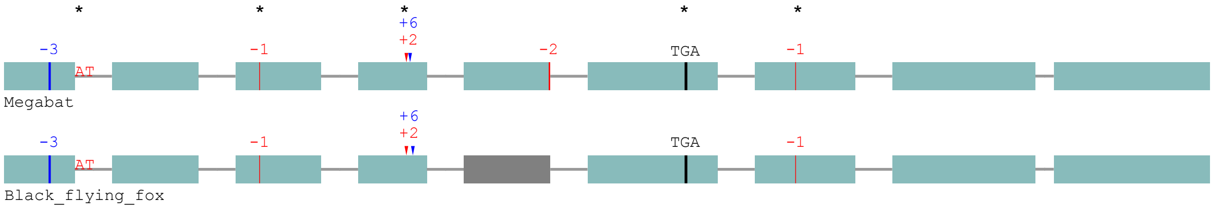

Supplementary Figure 31: Mutations in *APMAP* in the black flying fox and the large flying fox.

A number of shared inactivating mutations (asterisks) in different exons show that the loss of *APMAP* already happened in the ancestor of both fruit bats.

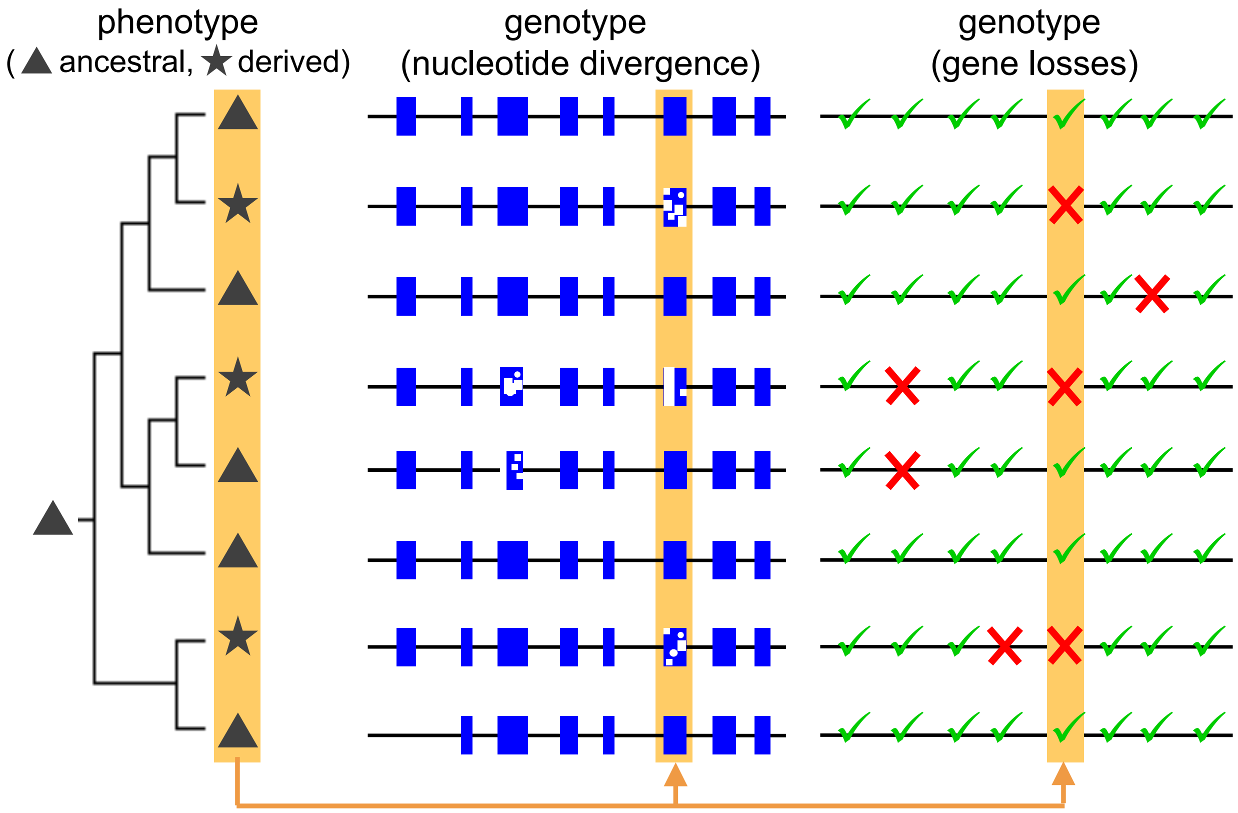

Supplementary Figure 32: Adopting the Forward Genomics framework to search for genes that are lost in independent lineages sharing the same phenotypic adaptation. Given an ancestral phenotype that has changed independently across species (left panel), the original Forward Genomics approach [5,6] searches genome-wide for a region with higher nucleotide divergence in all species with the derived phenotype (highlighted in orange; middle panel). Here, we searched for genes that are preferentially lost in species that share the derived phenotype (right panel, highlighted in orange).

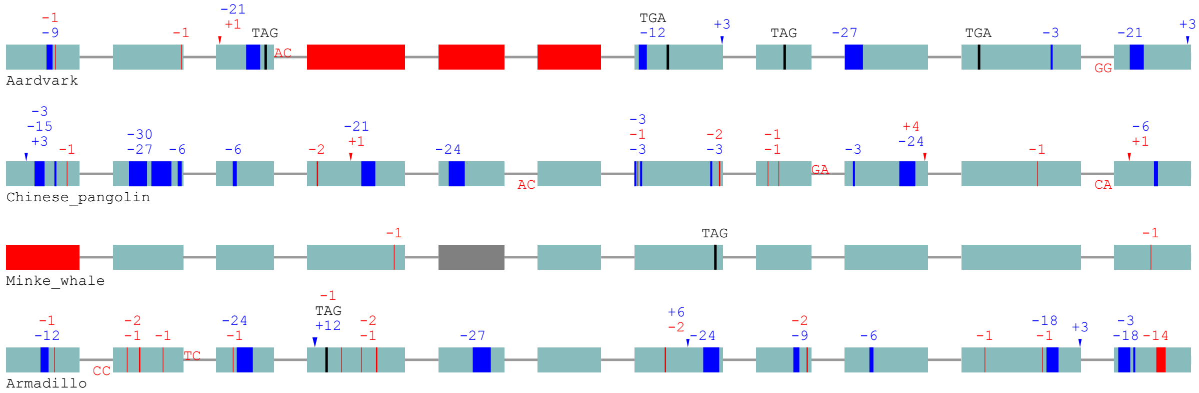

Supplementary Figure 33: Mutations in *ACP4* in species without tooth enamel.

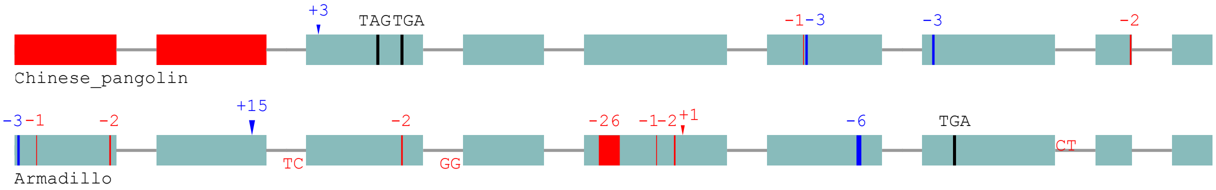

Supplementary Figure 34: Mutations in *DDB2* in pangolin and armadillo.
For pangolin, we estimated that *DDB2* was lost in the last ~5 Mya (Supplementary Table 5), which postdates the evolution of scales in this lineage. For armadillo, we estimated that *DDB2* was lost 84-92 Mya (Supplementary Table 5). This predates the oldest known fossil with preserved scales (Riostegotherium yanei, ~58 Mya [7]). However, it should be noted that the fossil record for this lineage is very sparse and lacks any fossils of basal armadillo and xenarthran species, thus it is unknown when scales evolved in the armadillo lineage.

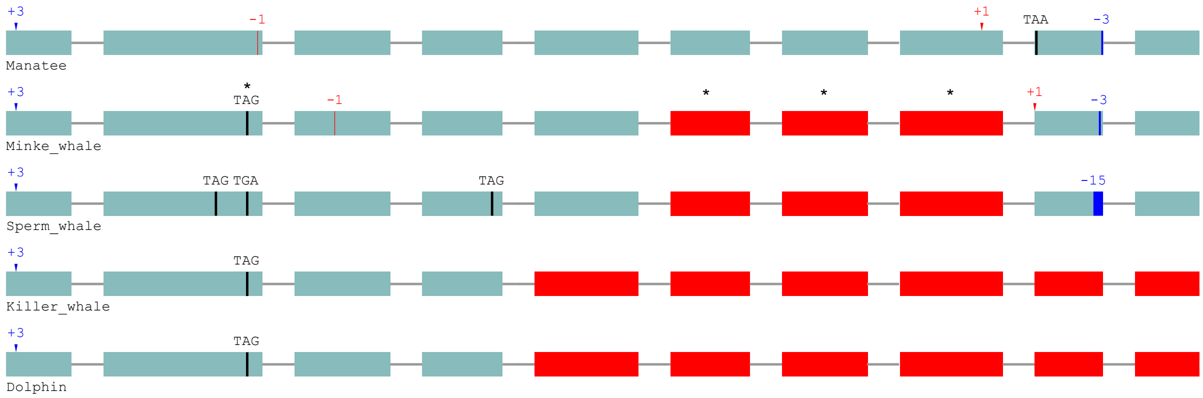

Supplementary Figure 35: Mutations in *MMP12* in cetaceans and manatee. *MMP12* has a stop codon (asterisk) that is shared between all four cetaceans. Also, the deletion of exons 6-8 (asterisk) has the same breakpoints in the minke and sperm whale. This shows that *MMP12* loss already happened in the cetacean ancestor before the split of the toothed and baleen whale lineage. For manatee, we estimated that *MMP12* loss happened at the base of this lineage (61-72 Mya), which predates the split of manatee and its fully-aquatic sister lineage dugong (26-53 Mya).

Step	Species	# mutations	# exons with mutations	# genes with mutations	% genes with mutations
0	Standard genome alignment without any filters and using longest transcript isoform				
	Mouse (mm10)	20770	8489	5705	42.3%
	Rat (rn5)	22675	10162	6327	46.9%
	Cow (bosTau7)	25020	10122	6502	48.2%
	Dog (canFam3)	24596	9956	6617	49.1%
1	Genome alignment after masking low quality bases and using longest transcript isoform				
	Mouse	20595	8420	5685	42.2%
	Rat	21750	9763	6161	45.7%
	Cow	21849	8871	5872	43.5%
	Dog	20442	8730	5959	44.2%
2	Genome alignment after filtering for assembly gaps and using longest transcript isoform				
	Mouse	20564	8392	5680	42.1%
	Rat	20097	8350	5696	42.2%
	Cow	20514	7714	5435	40.3%
	Dog	18866	7303	5227	38.8%
3	Genome alignment after excluding paralog and pseudogene alignments and using longest transcript isoform				
	Mouse	20382	8327	5655	41.9%
	Rat	19767	8276	5663	42.0%
	Cow	20418	7733	5461	40.5%
	Dog	18568	7255	5210	38.6%
4	Genome alignment and evaluating several transcript isoforms				
	Mouse	11387	5550	4092	30.3%
	Rat	11155	5653	4181	31.0%
	Cow	12073	5392	4072	30.2%
	Dog	10734	4956	3820	28.3%
5	Genome alignment after realigning with CESAR to exclude alignment ambiguities and splice site shifts, evaluating several isoforn				
	Mouse	2269	1580	1201	8.9%
	Rat	3065	2109	1662	12.3%
	Cow	2368	1558	1304	9.7%
	Dog	2099	1423	1178	8.7%
6	Genome alignment after excluding precise intron deletions, compensating frameshifts and U12 intron splice site mutations				
	Mouse	2016	1440	1132	8.4%
	Rat	2760	1920	1580	11.7%
	Cow	2163	1487	1254	9.3%
	Dog	1946	1374	1146	8.5%
7	Genome alignment after excluding mutations close to the protein's termini (within first/last 20% of the coding sequence)				
	Mouse	694	471	366	2.7%
	Rat	1103	730	627	4.6%
	Cow	802	530	465	3.4%
	Dog	665	452	368	2.7%

Analysis of the remaining inactivating mutations in presumably conserved genes

Species	no. genes with remaining mutations	genes with mutations in only 1 exon	
		number	percent
Mouse	366	311	85%
Rat	627	561	89%
Cow	465	423	91%
Dog	368	321	87%

Final number of false positives (inactivating mutations in multiple exons and < 60% intact reading frame)

Species	number	specificity
Mouse	32	99.76%
Rat	45	99.67%
Cow	41	99.70%
Dog	38	99.72%
**Average**	**39**	99.71%

Supplementary Table 1: Detailed breakdown of the specificity after each step in the gene loss detection pipeline and final specificity.

The underlying dataset is a list of 13,486 human genes that have annotated 1:1 orthologs in mouse (mm10), rat (rn5), cow (bosTau7) and dog (canFam3), and thus are likely conserved (Supplementary Data 1). The UCSC 100-way alignment [8] is used in step 0.

Assembly	Species	Scientific name
hg38	Human	Homo sapiens
panTro4	Chimp	Pan troglodytes
panPan1	Bonobo	Pan paniscus
ponAbe2	Orangutan	Pongo pygmaeus abelii
nomLeu3	Gibbon	Nomascus leucogenys
rheMac3	Rhesus	Macaca mulatta
macFas5	Crab-eating macaque	Macaca fascicularis
papAnu2	Baboon	Papio anubis
chlSab2	Green monkey	Chlorocebus sabaeus
rhiRox1	Golden snub-nosed monkey	Rhinopithecus roxellana
calJac3	Marmoset	Callithrix jacchus
saiBol1	Squirrel monkey	Saimiri boliviensis
tarSyr2	Tarsier	Tarsius syrichta
otoGar3	Bushbaby	Otolemur garnettii
speTri2	Squirrel	Spermophilus tridecemlineatus
jacJac1	Lesser Egyptian jerboa	Jaculus jaculus
micOch1	Prairie vole	Microtus ochrogaster
perManBai1	Prairie deer mouse	Peromyscus maniculatus bairdii
mesAur1	Golden hamster	Mesocricetus auratus
mm10	Mouse	Mus musculus
rn6	Rat	Rattus norvegicus
nanGal1	Upper Galilee mountains blind mole rat	Nannospalax galili
hetGla2	Naked mole-rat	Heterocephalus glaber
cavPor3	Guinea pig	Cavia porcellus
chiLan1	Chinchilla	Chinchilla lanigera
octDeg1	Brush-tailed rat	Octodon degus
oryCun2	Rabbit	Oryctolagus cuniculus
ochPri3	Pika	Ochotona princeps
vicPac2	Alpaca	Vicugna pacos
camFer1	Bactrian camel	Camelus ferus
turTru2	Dolphin	Tursiops truncatus
orcOrc1	Killer whale	Orcinus orca
phyCat1	Sperm whale	Physeter catodon
balAcu1	Minke whale	Balaenoptera acutorostrata scammoni
panHod1	Tibetan antelope	Pantholops hodgsonii
bosTau8	Cow	Bos taurus
bisBis1	Bison	Bison bison bison
capHir1	Domestic goat	Capra hircus
equCab2	Horse	Equus caballus
cerSim1	Rhinoceros	Ceratotherium simum
felCat8	Cat	Felis catus
canFam3	Dog	Canis lupus familiaris
musFur1	Ferret	Mustela putorius furo
ailMel1	Panda	Ailuropoda melanoleuca
ursMar1	Polar bear	Ursus maritimus
odoRosDiv1	Pacific walrus	Odobenus rosmarus divergens
lepWed1	Weddell seal	Leptonychotes weddellii
manPen1	Chinese pangolin	Manis pentadactyla
pteAle1	Black flying-fox	Pteropus alecto
pteVam1	Megabat	Pteropus vampyrus
eptFus1	Big brown bat	Eptesicus fuscus
myoDav1	Davids myotis bat	Myotis davidii
myoLuc2	Microbat	Myotis lucifugus
eriEur2	Hedgehog	Erinaceus europaeus
sorAra2	Shrew	Sorex araneus
conCri1	Star-nosed mole	Condylura cristata
loxAfr3	Elephant	Loxodonta africana
eleEdw1	Cape elephant shrew	Elephantulus edwardii
triMan1	Manatee	Trichechus manatus latirostris
chrAsi1	Cape golden mole	Chrysochloris asiatica
echTel2	Tenrec	Echinops telfairi
oryAfe1	Aardvark	Orycteropus afer afer
dasNov3	Armadillo	Dasypus novemcinctus

Supplementary Table 2: 62 placental mammals and their genome assemblies that were used in this study.

Gene	Lost in species	Reference
BEX5	mouse	Alvarez *et al.* 2005, ref [9]
MX1	dolphin, orca, sperm whale	Braun *et al.* 2015, ref [10]
MX2	dolphin, orca, sperm whale	Braun *et al.* 2015, ref [10]
PCSK9	cow	Cameron *et al.* 2008, ref [11]
BOK	dog	Derrien *et al.* 2009, ref [12]
PROZ	dog	Derrien *et al.* 2009, ref [12]
SERPINA10	dog	Derrien *et al.* 2009, ref [12]
ABCA4	blind mole rat	Fang *et al.* 2014, ref [13]
BEST1	blind mole rat	Fang *et al.* 2014, ref [13]
BFSP2	blind mole rat	Fang *et al.* 2014, ref [13]
CNGB3	blind mole rat	Fang *et al.* 2014, ref [13]
CRYBA1	blind mole rat	Fang *et al.* 2014, ref [13]
GUCY2F	blind mole rat	Fang *et al.* 2014, ref [13]
IMPG1	blind mole rat	Fang *et al.* 2014, ref [13]
PPEF2	blind mole rat	Fang *et al.* 2014, ref [13]
RBP3	blind mole rat	Fang *et al.* 2014, ref [13]
SLC24A1	blind mole rat	Fang *et al.* 2014, ref [13]
SNTN	old world monkeys	George *et al.* 2011, ref [14]
MLN	mouse, rat, guinea pig	He *et al.* 2010, ref [15]
MLNR	mouse, rat, guinea pig	He *et al.* 2010, ref [15]
ABCB4	guinea pig, horse	Hiller *et al.* 2012, ref [5]
TAS1R1	dolphin	Jiang *et al.* 2012, ref [16]
TAS1R2	dolphin	Jiang *et al.* 2012, ref [16]
TAS1R3	dolphin	Jiang *et al.* 2012, ref [16]
GJA10	vespertilionid bats	Shen *et al.* 2013, ref [17]
CASP14	cetaceans	Strasser *et al.* 2015, ref [18]
CCL8	rabbit	van der Loo *et al.* 2016, ref [19]
AOC3	rat	Zhang *et al.* 2003, ref [20]

Supplementary Table 3: Previously-known gene losses in non-human primates that were detected by our approach.

Gene symbol	Ensembl Gene Identifier	Ensembl transcript identifier *	Lost species
DSG4	ENSG00000175065	ENST00000308128	Dolphin, Killer whale, Sperm whale, Minke whale
DSC1	ENSG00000134765	ENST00000257198	Dolphin, Killer whale, Sperm whale, Minke whale
TGM5	ENSG00000104055	ENST00000349114	Dolphin, Killer whale, Sperm whale, Minke whale
GSDMA	ENSG00000167914	ENST00000301659	Dolphin, Killer whale, Sperm whale, Minke whale
ALOXE3	ENSG00000179148	ENST00000448843	Dolphin, Killer whale, Sperm whale, Minke whale
AMPD3	ENSG00000133805	ENST00000529834	Sperm whale
BCO1	ENSG00000135697	ENST00000258168	Sperm whale
SLC22A6	ENSG00000197901	ENST00000360421	Black flying fox, Large flying fox
SLC22A12	ENSG00000197891	ENST00000377572	Black flying fox, Large flying fox
SLC2A9	ENSG00000109667	ENST00000309065	Black flying fox, Large flying fox
RHBG	ENSG00000132677	ENST00000537040	Black flying fox, Large flying fox
AQP6	ENSG00000086159	ENST00000315520	Black flying fox, Large flying fox
MOGAT2	ENSG00000166391	ENST00000198801	Black flying fox, Large flying fox
FABP6	ENSG00000170231	ENST00000402432	Black flying fox, Large flying fox
SLC27A6	ENSG00000113396	ENST00000395266	Black flying fox, Large flying fox
APMAP	ENSG00000101474	ENST00000217456	Black flying fox, Large flying fox
FAM3B	ENSG00000183844	ENST00000398647	Black flying fox, Large flying fox
FFAR3	ENSG00000185897	ENST00000327809	Black flying fox, Large flying fox
ACP4	ENSG00000142513	ENST00000270593	Aardvark, Chinese pangolin, Minke whale, Armadillo
DDB2	ENSG00000134574	ENST00000378603	Chinese pangolin, Armadillo
MMP12	ENSG00000262406	ENST00000571244	Dolphin, Killer whale, Sperm whale, Minke whale, Manatee

Supplementary Table 4: Previously unknown gene losses that are discussed in the manuscript.

*: The exon-intron structure of this transcript is used to show the inactivating mutations in the Supplementary Figures 14-31 and 33-35.

Branch leading to	Gene	Ka/Ks value for entire branch (K) *	Ka/Ks value for species with an intact *gene* (K_s)	confidence interval for species divergence time (Mya, TimeTree)		Estimated time interval for *gene* loss (Mya)	
Toothed whale ancestor	DSC1	0.8923	0.2343	30.6	35.5	26.3	30.5
Minke whale	DSC1	1.0768	0.2364	30.6	35.5	30.6	35.5
Toothed whale ancestor	ALOXE3	0.6927	0.1213	30.6	35.5	19.9	23.1
Minke whale	ALOXE3	0.3467	0.1269	30.6	35.5	7.7	8.9
Sperm whale	AMPD3	1.2051	0.0819	31	37	31	37
Sperm whale	BCO1	1.2543	0.1956	31	37	31	37
Pangolin	DDB2	0.3638	0.3205	70	79	4.5	5.0
Armadillo	DDB2	0.9181	0.3211	96	105	84.4	92.3
Manatee	MMP12	1.3350	0.5386	61	72	61	72

Supplementary Table 5: Estimates of the gene loss dates.

* Ka/Ks values that exceed 1 indicate that the gene evolved neutrally along the entire branch, thus the loss likely happened at the base of the respective branch.

		Gene loss in				Number of species with data	
		without teeth/enamel		others			
Gene	P-value GLS	number of species	species	number of species	species	without teeth/enamel	others
ACP4 *	4.11E-16	3	Minke whale;Chinese pangolin;Aardvark	1 #	Polar bear #	4	47
MMP20 **	2.99E-15	2	Minke whale;Aardvark	0		3	52
ZADH2	3.16E-14	2	Aardvark;Armadillo	0		3	54
POLM	4.19E-14	2	Minke whale;Chinese pangolin	0		2	47
C4orf26 ***	4.29E-11	2	Minke whale;Chinese pangolin	0		4	56
OOEP	2.30E-07	2	Chinese pangolin;Aardvark	1	Bactrian camel	3	44

Supplementary Table 6: Forward Genomics hits for the enamel loss phenotype.

#: Assembly errors in polar bear. The assembly shows a partial deletion of the downstream half of exon 5 and the donor splice site. Searching polar bear SRA entries (accession number: SRX327134 and SRX327154) with the flanks of the "deletion" reveals no reads that span it. However, searching with the panda bear sequence covering the "deletion" yields several clear hits to polar bear reads, showing that this deletion is an assembly error. The second "mutation" in polar bear ACP4 is a 40 bp deletion in exon 10. Again, searching polar bear SRA entries with the flanks of the "deletion" reveals no reads that span it. In contrast, searching SRA with the Weddell seal sequence covering the "deletion" yields several clear hits (>95% identity) to polar bear genomic reads, showing that this deletion is an assembly error too.

*: Armadillo has lost *ACP4*, however we do not list it here, since the %intact reading frame of 68.6% is greater than our threshold of 60%.

**: For pangolin, we could not compute a %intact value, because the *MMP20* locus is not assembled on a single scaffold. However, reference [21] reports inactivating mutations in the pangolin. Armadillo has an intact reading frame, consistent with reference [21].

***: Aardvark and armadillo have an intact reading frame, consistent with reference [22].

		Gene loss in				Number of species with data	
		with scales		others			
Gene	P-value GLS	number of species	species	number of species	species	with scales	others
TMEM173	2.9E-43	2	Chinese pangolin;Armadillo	0		2	58
REP15	3.5E-08	2	Chinese pangolin;Armadillo	2	Guinea pig;Elephant	2	54
DDB2	2.1E-07	2	Chinese pangolin;Armadillo	1 #	Polar bear #	2	58

Supplementary Table 7: Forward Genomics hits for the scale phenotype.
#: Assembly error in polar bear. The assembly shows a partial deletion of the downstream half of exon 4 and the donor splice site. Searching polar bear SRA entries (accession number: SRX327134 and SRX327154) with the flanks of the "deletion" reveals no reads that span it. Searching with the giant panda sequence covering the "deletion" gives several polar bear reads that have 100% identity, showing that this deletion is an assembly error.

		Gene loss in				Number of species with data	
		fully-aquatic		others			
Gene	P-value GLS	number of species	species	number of species	species	fully-aquatic	others
SERPINB12	8.94E-15	2	Minke whale;Manatee	0		3	54
PON1	1.64E-13	5	Dolphin;Killer whale;Sperm whale;Minke whale;Manatee	1	Weddell seal	5	56
ANXA9	2.86E-12	5	Dolphin;Killer whale;Sperm whale;Minke whale;Manatee	0		5	50
TAS2R1	8.65E-12	3	Killer whale;Sperm whale;Manatee	2	Naked mole-rat;Star-nosed mole	3	51
MMP12	5.17E-11	5	Dolphin;Killer whale;Sperm whale;Minke whale;Manatee	0		5	53
GPR113	2.33E-10	5	Dolphin;Killer whale;Sperm whale;Minke whale;Manatee	1	Weddell seal	5	52
KRT82	1.83E-09	4	Dolphin;Sperm whale;Minke whale;Manatee	0		5	52
MSS51	9.98E-09	5	Dolphin;Killer whale;Sperm whale;Minke whale;Manatee	1	Armadillo	5	54
REG4	1.02E-08	3	Sperm whale;Minke whale;Manatee	2	Bushbaby;Horse	4	49
KLK8	6.13E-07	4	Dolphin;Killer whale;Minke whale;Manatee	0		5	54

Supplementary Table 8: Forward Genomics hits for the fully-aquatic phenotype

Supplementary Note 1

Cetacean-specific gene losses may contribute to hair loss and unique adaptations of the cetacean epidermis

The skin of cetaceans differs in many aspects from that of other mammals. The marine environment has a much greater density and viscosity, and exerts a greater pressure than a terrestrial environment [23]. A hallmark of adapting to the marine environment is the very thick cetacean epidermis, which can be 15 to 20 times thicker than in humans [24,25]. In contrast to other mammals, the epidermis consists of only three layers (stratum corneum, stratum spinosum, and stratum basale); the strata granulosum and lucidum are absent in cetaceans [23-26]. Furthermore, the cells in the stratum corneum often retain their nuclei (a condition known as parakeratosis), which is characteristic of an incomplete keratinization process [23,24,26]. Stratum corneum cells possess no keratohyalin granules and non-aggregated keratin filaments [27]. Measurements in the bottlenose dolphin have shown that the outermost cell layers are renewed 8.5 times faster than in humans, which helps to maintain a smooth surface and limits microbe colonization in the aquatic environment [28]. Furthermore, cetaceans have lost key mammal-defining characteristics such as hair and sebaceous and sweat glands [24,25]. Hair or fur in a fully-aquatic animal cannot provide thermal insulation but would increase drag and thus slow down locomotion, indicating that hair loss is likely adaptive [24].

Previous studies reported that cetaceans have an increased pseudogenization rate in hair keratin and keratin-associated protein (*KRTAP*) genes [29,30] and relaxed selection in the hair follicle gene *HR* (HR, lysine demethylase and nuclear receptor corepressor) [31]. These findings provide genomic signatures associated with hair loss. In addition, a previous study [18] identified the complete deletion of the epidermal protease *CASP14* that is involved in terminal keratinocyte differentiation. Since the knockout of *CASP14* does not cause parakeratosis but makes mice prone to developing acetone- or imiquimod-induced parakeratosis [32], *CASP14* loss in cetaceans is likely a consequence of the incomplete keratinization process that results in their parakeratotic stratum corneum.

Here, we largely extend previous findings by identifying several gene losses in cetaceans that could be causally involved in hair loss and other key aspects of the cetacean epidermis:

- *DSG4* (desmoglein 4)
- *DSC1* (desmocollin 1)
- *TGM5* (transglutaminase 5)
- *GSDMA* (gasdermin A).

Shared inactivating mutations show that the loss of *DSG4*, *TGM5* and *GSDMA* already happened before the split of the toothed and baleen whale lineage. We further estimated that the loss of *DSC1* overlaps the split of the cetacean ancestor (Supplementary Table 5). Thus, the loss of these genes coincides with a period during which epidermal adaptations evolved in cetaceans. In addition, we report the cetacean-specific loss of *ALOXE3* (arachidonate lipoxygenase 3), which happened much later, after the split of the baleen and toothed whale lineage (Supplementary Table 5). This suggests that *ALOXE3* loss is a consequence of already existing epidermal adaptations, as described below.

Adhesion of epidermal cells is partially mediated by desmosomes, which are specialized cell-cell junctions consisting of the cadherin family members desmogleins and desmocollins. Compared to other desmogleins and desmocollins, both *DSG4* and *DSC1* are specifically localized to the highly differentiated, upper epidermis layers in human [33,34], and strongly bind to each other [35]. In addition, both proteins are expressed in the hair follicle, localized to the inner root sheath cuticle and other areas, where they are involved in keratinocyte cell adhesion [33,36]. Mutations in human and mouse *DSG4* cause hypotrichosis (loss or reduction of hair) and hyperkeratosis (thicker stratum corneum) [37-40]. *DCS1* knockout in mice also causes hyperkeratosis, increased transepidermal water loss, and alopecia (hair loss) [41]. The loss of two desmosome components that are specifically expressed in the upper-most epidermis layers likely explains the reduced desmosome number in the stratum corneum of cetaceans [26], which would provide a mechanism that could be causally involved in the fast shedding rates of stratum corneum cells observed in dolphin [28]. Furthermore, the knockout phenotypes of *DSG4* and *DSC1* suggest that the loss of these genes could have contributed to the loss of hair in cetaceans.

TGM5 encodes a transglutaminase expressed in the stratum spinosum and stratum granulosum, playing a role in the terminal differentiation of keratinocytes by cross-linking structural corneocyte proteins such as loricrin and involucrin [42,43]. Loss-of-function mutations in human *TGM5* are associated with peeling skin syndrome, which involves shedding of the outer layers of the epidermis [44]. Thus, in addition to the loss of *DSG4* and *DSC1*, the loss of *TGM5* could have contributed to the increased shedding rates of stratum corneum cells.

In contrast to other mammals where *GSDMA* is a single-copy gene, mice have three *GSDMA* genes. Of these, *GSDMA3* is well studied and is expressed in the suprabasal epidermis layers, in hair follicles, and in the sebaceous gland [45,46]. Strikingly, despite the existence of two additional *GSDMA* genes in mouse, mutations in *GSDMA3* alone cause alopecia, a thicker epidermis and dermis, and absent hair follicle-associated sebaceous

glands [45,46]. Since cetaceans exhibit only one *GSDMA* copy that was clearly lost in the cetacean ancestor (evident from a shared partial deletion), the loss of *GSDMA* may have a causal role in several skin phenotypes observed in cetaceans.

ALOXE3 is expressed in the stratum granulosum in the skin [47]. *ALOXE3* knockout in mice leads to an altered ceramide composition in the stratum corneum and a 4-fold increased transepidermal water loss [47]. Thus, *ALOXE3* loss could be related to skin barrier changes as the cetacean stratum corneum contains little ceramides [26]. Consistent with the fact that mutations in human *ALOXE3* are associated with recessive congenital ichthyosis [48], *ALOXE3* knockout in mice also results in hyperkeratosis [47], a hallmark of ichthyosis. However, in contrast to *DSG4*, *DSC1*, *TGM5*, and *GSDMA*, the loss of *ALOXE3* – while specific to cetaceans – happened after the split of the baleen and toothed whale lineage (Supplementary Table 5). Thus, the loss of *ALOXE3* likely happened as a consequence of epidermal adaptations in cetaceans.

Overall, we identified a suite of genes whose loss provides mechanistic explanations for several aspects of the unique cetacean skin morphology that exhibits a thicker epidermis (*DSG4*, *DSC1*, *GSDMA*), increased shedding of stratum corneum cells (*DSG4*, *DSC1*, *TGM5*), no hair (*GSDMA*, *DSG4*, *DSC1*) and no sebaceous glands (*GSDMA*). In contrast to the terrestrial environment, where the loss of two of these genes (*DSC1* and *ALOXE3*) would be detrimental due to increased transepidermal water loss, the loss of these genes was likely permitted in the aquatic environment where hydration is less important. In summary, our results suggest that the loss of several genes could have played a causal role in the adaptation of the cetacean skin to the aquatic environment.

The manatee is another lineage that independently evolved a fully-aquatic lifestyle. We found that all five genes are intact in the manatee. Compared to cetaceans, the manatee skin is far less studied. Epidermal characteristics that are shared between manatees and cetaceans are the absence of hair, an increased stratum corneum thickness and a three-layer epidermal structure [49]. This suggests that genomic changes other than the described gene losses are responsible for these epidermal changes in the manatee. In contrast to cetaceans, the manatee epidermis exhibits fully differentiated, anucleated keratinocytes [50]. Furthermore, while dolphins have a high shedding rate of cells in the stratum corneum, manatees likely exhibit a rather low shedding rate, since their skin is often covered with algae and other microbes. This may explain why genes such as *DSG4*, *DSC1* and *TGM5* are not lost in the manatee.

Supplementary Note 2

Loss of *AMPD3* in the sperm whale as a potential adaptation to long dives

AMPD3 encodes an enzyme that deaminates adenosine monophosphate (AMP) to inosine monophosphate (IMP). Erythrocytes only express *AMPD3*, in contrast to other tissues that also express *AMPD1* and *AMPD2* [51,52]. Since erythrocytes cannot convert IMP back to AMP and the adenylate equilibrium maintains a ratio of ATP:ADP:AMP of ~100:10:1, the activity of AMPD3 results in a shrinking adenine nucleotide pool and reduced ATP levels. The function of AMPD3 as a key regulator of the adenine nucleotide pool size in erythrocytes is evident from *AMPD3* knockout mice that are phenotypically normal but have a 3-fold higher level of ADP and ATP in erythrocytes [51-53]. Likewise, AMPD3 deficiency in human also increases the ATP level in erythrocytes [54].

Since ATP is an allosteric effector that stabilizes O_2-unloaded hemoglobin in vertebrates [55,56], erythrocytes in *AMPD3* knockout mice have a half-saturation pressure of oxygen that is significantly increased by 4 to 6 mmHg (a higher pressure is needed to reach 50% oxygen saturation of hemoglobin), shifting the oxygen–hemoglobin dissociation curve to the right [52]. Consistent with a role of organic phosphates such as ATP in regulating the hemoglobin affinity for oxygen, the observed right shift in *AMPD3* knockout mice can primarily be attributed to the increased erythrocyte ATP level [52].

We detected the loss of the *AMPD3* gene only in the sperm whale, which is one of the deepest and longest diving mammals. While most cetaceans, pinnipeds and the manatee typically dive for less than 5 minutes, the sperm whale routinely dives for 40-60 min to depths of 400-900 meters [57,58]. The right-shift of the oxygen–hemoglobin dissociation curve observed in *AMPD3* knockout mice is likely adaptive for a mammal with long diving times. In contrast to a left-shift, commonly observed as a high-altitude adaptation [59], a right-shift results in a reduced affinity of hemoglobin for O_2, which facilitates O_2 release from erythrocytes to the tissue. The sperm whale inhales at sea level, where the flat part of a right-shifted dissociation curve should still allow for near 100% O_2 saturation (Figure 3). However, at the end of long dives, the tissue is O_2 depleted, resulting in a low partial O_2 pressure in the capillaries. Under these conditions, a right-shifted curve improves O_2 delivery from the partially-saturated hemoglobin to the tissue.

Remarkably, a right-shifted dissociation curve was also observed for the crocodile hemoglobin. Crocodiles are able to stay submerged for over an hour. However, they evolved a completely different mechanism as their hemoglobin is insensitive to allosteric

effectors such as ATP and 2,3BPG [60]. Instead, a minimum of 12 amino acid changes enabled two bicarbonate ions derived from CO_2 to bind to deoxyhemoglobin [60-62]. Thus, in crocodiles, the end-product of oxidative metabolism is directly coupled to O_2 release. Together, this suggests that a right-shifted dissociation curve is generally adaptive for long-diving species and indicates that *AMPD3* loss in the sperm whale contributes to its exceptional diving ability.

Supplementary Note 3

Loss of BCO1, a key enzyme for vitamin A synthesis, is likely a consequence of the beta-carotene poor diet of sperm whales

We found that the sperm whale has lost the *BCO1* (Beta-Carotene Oxygenase 1) gene, which is present in other cetaceans and other mammals. *BCO1* encodes an enzyme that catalyzes the cleavage of beta-carotene into two retinal molecules (a form of vitamin A) [63]. The loss of *BCO1* provides new insights into the metabolism of the sperm whale by implying that this species is unable to convert carotenoids into vitamin A.

The diet of sperm whales consists predominantly of medium-sized squid, which contain no or very little beta-carotene but contain larger amounts of vitamin A [64-66]. This suggests that sperm whales obtain their vitamin A supply directly from their diet. Consequently, the high vitamin A concentration observed in sperm whale blubber and liver [67] is likely of dietary rather than synthesized origin. In contrast, the minke whale that feeds on carotene-rich krill [67] has an intact *BCO1* gene. Hence, the loss of *BCO1* is likely a consequence of relaxed selection on an enzyme whose substrate (beta-carotene) is scarce while its product (vitamin A) is present in large amounts in the diet.

Supplementary Note 4

Gene losses may contribute to renal adaptations in fruit bats

Frugivorous bats typically chew fruits to extract the fruit juice and spit out the pulp. To satisfy the high energy demands of powered flight, these bats consume large amounts of fruit (sometimes more than their own body weight every night [68,69]), resulting in a large amount of ingested juice. To excrete the excess dietary water, they produce large amounts of a very dilute urine [70]. The kidney of fruit bats shows morphological modifications such as a decreased relative thickness of the medulla that reduce the urine concentrating ability [71-73].

We found that both frugivorous bats in our dataset, the black flying fox and large flying fox, have lost several genes that are specifically expressed in the kidney and are involved in renal secretion and reabsorption processes:

- *SLC22A6* (solute carrier family 22 member 6) gene encoding the Organic Anion Transporter OAT1
- *SLC22A12* (solute carrier family 22 member 12) encoding URAT1
- *SLC2A9* (solute carrier family 2 member 9) encoding GLUT9
- *RHBG* (Rh family B glycoprotein)
- *AQP6* (aquaporin 6).

As detailed below, these gene losses suggest that not only the morphology, but also the physiology of the kidney adapted to the challenges imposed by the frugivorous diet.

OAT1 (encoded by *SLC22A6*) is highly expressed in the kidney and is localized at the basolateral membrane in proximal tubule cells [74-76]. OAT1 plays a major role in the tertiary active transport of organic anions from blood to urine by mediating the basolateral uptake step [77]. *OAT1* knockout mice excrete 30% less urate in urine and show decreased renal secretion of endogenous organic anions [77,78]. Given the large amount of urine that fruit bats produce, the loss of this gene may be beneficial for preserving organic anions.

Furthermore, we found that fruit bats are “double knockouts” for the two cooperating urate reabsorbing transporters URAT1 (encoded by *SLC22A12*) and GLUT9 (encoded by *SLC2A9*), and appear to maintain only the two low affinity transporters OAT4 (*SLC22A11*) and OAT10 (*SLC22A13*) [79,80] that were classified as intact genes in our analysis. *SLC22A12* is expressed in the apical membrane of proximal tubule cells and transports urate from the lumen into the cell in exchange for organic anions to maintain electrical balance [78,81]. Loss-of-function mutations in *SLC22A12* in both human and mouse impair

urate reabsorption [78,81]. Importantly, the urine of knockout mice exhibits a reduced metabolite concentration [78]. The second gene, *SLC2A9,* is expressed in liver, kidney, and placenta [82] and also functions as a urate transporter [83]. The *SLC2A9* gene has two alternative transcription start sites and produces two transcripts that differ in their N-terminus. In cells of the proximal tubule and distal convoluted tubule, the long GLUT9 protein is targeted to the basolateral membrane, while the short GLUT9 protein is targeted to the apical membrane [82,84]. GLUT9 cooperates with URAT1 in renal urate reabsorption and transports it across both the apical and basolateral membrane [84,85]. GLUT9 knockout mice have drastically increased urate excretion, two-fold increased water intake and a urine osmolality reduced to ~25% of that of wildtype mice [84]. Strikingly, the reduced urine osmolality is not primarily explained by increased water intake since knockout mice subjected to water deprivation show the same reduction in urine osmolality [84]. Hence, together with the loss of OAT1 and URAT1 that reduces urinary secretion of metabolites and organic anions, the urinary concentrating defect resulting from GLUT9 loss likely helps fruit bats to dilute their urine. Our findings suggest that loss of key renal transporters could be an evolutionary mechanism to adapt to a water-rich frugivorous diet.

RHBG (synonym *SLC42A2*) encodes an electroneutral ammonium/proton exchanger that is expressed in the kidney, liver and skin [86]. In the kidney, *RHBG* is expressed at the basolateral membranes of epithelial cells of the connecting segment, the collecting tubule and cortical collecting duct [87], where it contributes to renal ammonium excretion under both basal conditions and during metabolic acidosis [88]. Since the amount of protein intake is a major factor influencing the production of endogenous acids [89], the role of *RHBG* in regulating acid-base homeostasis [88] is likely less important for fruit bats with their protein-poor diet. For the following two reasons, the loss of *RHBG* could be beneficial for fruit bats. First, reduced ammonium excretion reduces urinary nitrogen loss, which might be an advantage for species with a protein-poor diet. Second, ammonia produced by proximal tubule cells and secreted into the lumen acts as an intrarenal, paracrine signaling molecule that inhibits potassium secretion and sodium reabsorption in the cortical collecting duct [90]. This is relevant for fruit bats as their diet has a high potassium but low sodium content. Consequently, fruit bats are able to efficiently excrete potassium (two-fold higher levels compared to non-frugivorous bats) while preserving precious sodium [91]. The loss of RHBG, an ammonium secreting protein that is expressed in the distal kidney segments where most ammonia excretion takes place, suggests that reduced ammonium secretion could be a causal mechanism that help bats to efficiently excrete potassium and reabsorb sodium.

Fruit bats have lost the *AQP6* gene that is the only known aquaporin that does not function as a water channel but rather as an anion channel. *AQP6* is specifically expressed in the

kidney. The encoded transmembrane protein localizes to intracellular vesicles in epithelial cells of the glomerulus, proximal tubules and collecting duct, but not to apical or basolateral membranes [92]. While other aquaporins are impermeable to ions, the AQP6 anion channel has high nitrate permeability [93,94] due to a single amino acid difference to other aquaporins [95]. The physiological function of *AQP6* has not been well characterized; however, it is interesting to note that *AQP6* is upregulated by chronic metabolic alkalosis and increased water intake [96], which provides a putative link to the high amount of water that fruit bats consume daily.

Supplementary Note 5

Gene losses can be a consequence and may contribute to metabolic adaptations in fruit bats

The diet of frugivorous bats contains predominantly sugars and very little fat and protein [97,98]. The metabolism of these bats has adapted to using sugars as the major energy source [69,99,100]. Facilitated by paracellular transport, frugivorous bats are able to absorb almost all of the sugar ingested within 45 min after a meal, and they switch extremely rapidly (within minutes) to metabolizing the ingested dietary sugar [69,99,100].

We identified a number of metabolism-related genes that are specifically lost in both frugivorous bats (black flying fox and large flying fox) present in our dataset. Some of these losses are likely a consequence, while others could be causally involved in metabolic adaptations to their sugar-rich and fat-poor diet:

- *MOGAT2* (monoacylglycerol O-acyltransferase 2)
- *FABP6* (fatty acid binding protein 6) encoding the ileal lipid binding protein ILBP
- *SLC27A6* (solute carrier family 27 member 6) encoding the fatty acid transport protein 6 (FATP6)
- *APMAP* (adipocyte plasma membrane associated protein)
- *FAM3B* (family with sequence similarity 3 member B) encoding the pancreatic-derived factor (PANDER)
- *FFAR3* (free fatty acid receptor 3).

The frugivorous bats are the only mammals in our dataset that have lost the *MOGAT2* gene. *MOGAT2*, which is predominantly expressed in the small intestine in mice [101,102], plays a major role in the absorption of dietary fat. To absorb fat in the intestine, triacylglycerol is broken down into monoacylglycerol and fatty acids, which can enter the enterocytes of the small intestine, where triacylglycerol is resynthesized. MOGAT2 catalyzes the first step, which is the synthesis of diacylglycerol from 2-monoacylglycerol and fatty acyl-CoA [102]. The loss of this enzyme is likely a consequence of the fat-poor diet of frugivorous bats.

Fruit bats have lost the *FABP6* gene, which encodes ILBP (ileal lipid binding protein) that binds bile acids with a high affinity and fatty acids with a lower affinity. This gene is expressed in the enterocytes of the ileum, the final section of the small intestine [103]. The ileum has a key role in enterohepatic circulation by absorbing bile acids that aid in fat digestion in the intestine. Absorbed bile acids are then transported to the portal vein for

recycling in the liver. ILBP facilitates transcellular bile acid transport, which is important for efficient bile acid transport from the intestine to the portal blood [104]. As the frugivorous diet is poor in fat, it is likely that fruit bats experienced a reduced selective pressure to maintain this gene and consequently lost it.

Fruit bats have lost the *SLC27A6* gene, which encodes the heart-specific long-chain fatty acid transporter FATP6 that is localized to the sarcolemma of cardiac myocytes [105]. In human, beta-oxidation of fatty acids provides 67% of the energy for cardiac myocytes; glucose and lactate are the other energy sources [106]. Since the fruit bat diet consists mostly of simple carbohydrates and these bats are able to maintain high glucose levels even when fasting, the loss of FATP6 likely reflects the diminished dependence on fatty acids for providing energy to cardiac myocytes. This gene loss is therefore likely a consequence of adapting to the frugivorous diet and provides new insights into the metabolism of the heart by indicating that sugars replaced fatty acids as the major energy source in this organ.

APMAP encodes a transmembrane protein that is highly expressed in adipocytes [107]. During adipocyte differentiation, *APMAP* is upregulated more than 13-fold [107,108]. APMAP is a direct target of PPARγ, which is considered to be the master regulator of adipogenesis [108]. Short hairpin RNA mediated silencing of *APMAP* during adipocyte differentiation decreases the expression of adipogenesis marker genes and results in differentiated cells that accumulate 80% less triglycerides, showing that APMAP is required for adipocyte differentiation [108]. Fatty acids contain a high energy density as hydrophobic fatty acids provide 10-times the energy per unit wet mass than hydrophilic glycogen (consisting of chains of glucose) [109]. Thus, triglycerides stored in adipocytes are a weight-saving energy source. However, in contrast to insectivorous bats that use fatty acids provided in their diet to replenish their fat reserves [110], fruit bats obtain very little fat with their diet and would have to convert dietary sugars into lipids. This conversion results in a 15% loss of energy compared to directly using glucose as fuel [109], which can explain why fruit bats power their flight activities preferentially with ingested sugars instead of relying on endogenous energy sources [69,99,100]. Since the generation of larger fat reserves is energetically costlier for fruit bats, bats use their rather small fat depots to meet energy requirements for the inactive period during the day and the first flight activity at the beginning of the night [99,100]. Fruit bats also turnover fat very rapidly and replace ~50% of their fat reserves in a single day [69,111]. Thus, the loss of *APMAP* is likely related to the absence of large fat depots and the rapid fat turnover in fruit bats.

In addition to these gene losses that are likely a consequence of dietary specialization, fruit bats have lost two genes involved in insulin metabolism and signaling (*FAM3B* and

FFAR3) that may contribute to metabolic adaptations to their sugar-rich diet. Out of all analyzed placental mammals, these genes are only lost in fruit bats, with the exception of cetaceans that also lost *FFAR3*. *FAM3B* encodes the pancreatic-derived factor (PANDER). PANDER is a cytokine whose expression in pancreatic beta-cells can be induced by glucose [112] and beta-cells co-secrete PANDER with insulin in a glucose-concentration dependent manner [113]. Overexpressing PANDER in the endocrine pancreas of mice results in impaired glucose tolerance and decreased hepatic insulin sensitivity, thus revealing a role of PANDER in regulating the insulin response of the liver [114]. Consistently, PANDER knockout mice show enhanced glucose tolerance and increased hepatic insulin sensitivity [115]. Thus, the loss of *FAM3B* in fruit bats could be a mechanism that facilitates hepatic processing of ingested sugar. *FFAR3* encodes a G protein-coupled receptor that is expressed in different tissues including pancreatic beta-cells [116]. *FFAR3* knockout in mice increases insulin secretion in a glucose-dependent manner, establishing FFAR3 as an inhibitor of insulin secretion [116,117]. The loss of *FFAR3* may contribute to the ability of fruit bats to secrete significantly more insulin (16% of the total insulin islet content) than mice, rats or human (1–2%) [118].

Supplementary Note 6

Forward Genomics: Genes lost in mammals without teeth or without enamel

We first applied our adopted Forward Genomics method to search for gene losses associated with the loss of enamel in aardvarks and armadillos and in the tooth-less minke whales and pangolins (Supplementary Table 6). We required that genes are lost in at least two of these four species. However, we did not consider genes lost exclusively in armadillo and pangolin as these animals have scales and shared gene losses could be associated to with the scale phenotype (see below). While the sperm whale (*Physeter macrocephalus*) has apparently enamel-coated teeth, these animals do not rely on their teeth for feeding, but ingest prey by suction [119]. This suggests that teeth in sperm whales are under relaxed selection, which is supported by the finding that the closely related adult pygmy sperm whale has enamel-less teeth [21]. Since the sperm whale could obscure this Forward Genomics search, we excluded this species from the analysis.

Using Forward Genomics, we identified two tooth-specific genes that play key roles in enamel formation and whose loss in enamel-less species has been described before [21,22,120], which serves as a positive control:

- *MMP20* (matrix metallopeptidase 20, also called enamlysin), a protease involved in degrading enamel matrix proteins during amelogenesis,
- *C4orf26* (chromosome 4 open reading frame 26), a gene associated with abnormal enamel formation (amelogenesis imperfecta) [121].

In addition to these known genes associated with the loss of enamel, we identified a new gene, *ACP4* (acid phosphatase 4), which is lost in aardvark, pangolin, armadillo and minke whale, but not in any of the other considered mammals. *ACP4* is expressed in secretory-stage ameloblasts, odontoblasts, and osteoblasts in developing molars of mice [122]. Mutations in *ACP4* have been linked to the tooth enamel disorder amelogenesis imperfecta, strongly indicating that *ACP4* has an important function in amelogenesis [122]. This example highlights that a Forward Genomics screen can uncover additional tooth-related genes lost in enamel- and tooth-less mammals that are missed by candidate gene studies.

Supplementary Note 7

Forward Genomics: Genes lost in mammals with scales

We searched for genes that are specifically lost in the two placental mammals that have body armor in the form of scales, the Nine-banded armadillo (*Dasypus novemcinctus*) and the Chinese pangolin (*Manis pentadactyla*) (Supplementary Table 7). It is important to note that the scales of pangolin and armadillo have different developmental origins and different histological properties. Pangolin scales are composed of non-mineralized keratins, whereas armadillo scales (also known as scutes) are composed of osteoderms (bony deposits in the dermis) [123]. Thus, it is unlikely to find gene losses that could play a causal role in scale development, however our Forward Genomics search may identify genes lost as a consequence of scale evolution, which could highlight hitherto unknown characteristics of scaly mammals.

We detected the loss of *DDB2* (damage specific DNA binding protein 2) in both pangolin and armadillo, but not in any other mammal in our dataset. *DDB2* has a key role in the repair of UV light-induced DNA damage. As part of the UV DNA-damage binding (UV-DDB) complex, DDB2 binds UV light-induced pyrimidine dimers, separates the damaged and undamaged strands and triggers nucleotide excision repair [124,125]. Mutations in *DDB2* cause xeroderma pigmentosum [126], an autosomal recessive disease characterized by hypersensitivity to sunlight, premalignant skin lesions and a high risk for skin cancer. Consistently, *DDB2* knockout mice are highly susceptible to UV-induced skin cancer [127,128]. Given that the lifespan in captivity is 12-15 years for armadillos and up to 20 years for pangolins, it is unlikely that the loss of a major component of the UV light-induced DNA damage repair machinery can be explained by a short lifespan. A possible explanation is that the scales covering the sun-exposed dorsal parts of the skin in armadillos and pangolins provide sufficient protection from UV light-induced DNA damage, which would imply that the loss of *DDB2* as a consequence of scale evolution is not deleterious for both scaly mammals. This assumption is supported by estimates of the gene loss dates (Supplementary Table 5), which indicates that *DDB2* loss in pangolin happened relatively recently (~5 Mya) after the evolution of scales (the oldest fossil with scales, Eomanis waldi [129], lived ~48 Mya).

The loss of *DDB2* raises the question whether other proteins of the global genome nuclear excision repair (GG-NER) mechanism were lost also in scaly mammals. Therefore, we investigated the genes whose encoded proteins interact with DDB2 based on the STRING database [130] and proteins that have been implicated in the function of the UV-DDB

complex [124]: *DDB1, CUL4A, CUL4B, XPC, RBX1, COPS2, COPS4, COPS5, COPS6, COPS8, RAD23B, ERRC1, ERRC2, ERRC3, ERRC4, ERRC5* and *RPA1*. In contrast to *DDB2*, we did not find any inactivating mutations in these genes in the pangolin and armadillo genome. A likely reason for preserving these genes is that they also function in repairing DNA lesions other than those caused by UV light, and they can have other unrelated functions. For example, while DDB1, CUL4A and XPC have a role in nuclear excision repair [131], these proteins also play important roles in post-translational and epigenetic gene regulation of stem cells and during embryogenesis [132,133].

Interestingly, we found that *DDB2* is intact in four subterranean mammals (the blind mole rat, naked mole rat, star-nosed mole, and cape golden mole) that live mostly in a dark underground environment. However, even species like the blind mole rat come to the surface for collecting hay for nest building, finding mating partners or during events like flooding [134]. These activities also take place during the day, which explains why the blind mole rat pelage matches the soil color and why this species makes up a substantial portion of the diet of diurnal birds of prey [134]. The star-nosed mole also often forages at the surface, both during day and night. Sunlight exposure during occasional activities outside of their tunnels may explain why *DDB2* is intact in these species.

Supplementary Note 8

Forward Genomics: Genes lost in fully-aquatic mammals

To identify gene losses that could be related to aquatic adaptations, we used Forward Genomics to search for genes that are lost in fully-aquatic mammals compared to terrestrial mammals and the semi-aquatic pinnipeds (Supplementary Table 8). Fully-aquatic mammals comprise two independent lineages: the cetaceans (represented by the genomes of dolphin, killer whale, sperm whale and minke whale) and the sirenia (manatee). Candidate genes were required to be lost in both independent lineages. This search retrieved the loss of *KRT82* (keratin 82), a type II hair keratin, and *KLK8*, a gene loss that correlates with skin and neuroanatomical differences of aquatic mammals [135]. In addition, we detected the loss of *MMP12* (matrix metallopeptidase 12).

MMP12 is only lost in the four cetacean species and the manatee. *MMP12* encodes a protease that is predominantly expressed by macrophages [136]. MMP12 degrades extracellular matrix proteins [136] and has a role in anti-viral immune defense [137]. The loss of *MMP12* in cetaceans and manatees could relate to *MMP12's* potent elastase activity, which degrades elastin in the extracellular matrix and is the main factor regulating elastin levels [136,138,139]. Elastin is the major component of elastic fibers that affect the biomechanical properties such as elasticity or resilience of several tissues including arteries, the lung, liver and skin. While the elastase activity of the related matrix metalloproteases MMP9 and MMP2 has also been implicated in arterial stiffening [140], a recent study investigating elastin degradation in *MMP12* knockout mice identified MMP12 as the main elastase for regulating chronic arterial stiffening [141]. Consistently, further experiments demonstrated that MMP12 degrades elastin more efficiently than MMP9 in the aorta and that only MMP12, but not MMP9, is able to degrade insoluble elastin fibers in the lung [142]. These findings establish MMP12 as the major elastase in the lung. The elastase activity of macrophage-secreted MMP12 in the lung is a key step in the pathogenesis of chronic obstructive pulmonary disease (COPD) [143]. COPD is mainly linked to cigarette smoking, which causes the recruitment of macrophages. Recruited macrophages then secrete MMP12, which leads to an increased elastase activity in the lower airways. The resulting degradation of elastin impairs the elasticity of alveoli, which contributes to a decreased expiratory airflow in COPD patients and leads to an incomplete emptying of the lungs. Fragments from degraded elastin are chemotactic for monocytes, which leads to a positive feedback loop by recruiting additional macrophages. *MMP12* knockout mice subjected to cigarette smoke do not exhibit this positive feedback loop, which in turn prevents destruction of bronchiolar and alveolar walls [144,145]. Consistent with

MMP12's role in COPD development, a SNP that decreases *MMP12* promoter activity reduces the risk of COPD [146].

We estimated that *MMP12* loss predates the split of manatee and its fully-aquatic sister lineage (the dugong), and predates the split of the fully-aquatic toothed and baleen whale lineages (Supplementary Figure 35, Supplementary Table 5). Thus, the loss of this gene coincided with a period during which adaptations to the aquatic environment evolved. The loss of *MMP12* and its elastin degrading function in the lung may contribute to a unique breathing adaptation of cetaceans and manatees: both lineages exhale very quickly and renew ~90% of the air in a single breath [147-149]. This is in stark contrast to terrestrial mammals that can renew only ~10% of the air. In cetaceans, this process is so fast that even blue whales with a 1,500 liter lung volume exhale and inhale in only 2 seconds [148]. Rapid exhalation is facilitated by extensive elastic tissue in their lungs that permits a greater expansion during inhalation and whose elastic recoil helps to empty the lungs quickly [149]. Consistent with *MMP12* being lost only in cetaceans and manatees but not in pinnipeds, many pinniped species exhale before diving and breath repeatedly after a dive [148,149]. Thus, the loss of the elastin-degrading *MMP12* may contribute to the higher elasticity of lung tissue in cetaceans and manatees. Higher elasticity facilitates rapid and explosive air exchange in these lineages, which is advantageous by clearing remaining water above the airways before inhaling and minimizing time spend at the surface during swimming, where wave drag dominates the total drag when swimming at >5 km/h [147,150].

Supplementary References

1 Sharma, V., Elghafari, A. & Hiller, M. Coding exon-structure aware realigner (CESAR) utilizes genome alignments for accurate comparative gene annotation. *Nucleic Acids Res* **44**, e103, doi:10.1093/nar/gkw210 (2016).

2 MacArthur, D. G. *et al.* A systematic survey of loss-of-function variants in human protein-coding genes. *Science* **335**, 823-828, doi:10.1126/science.1215040 (2012).

3 Chou, H. H. *et al.* Inactivation of CMP-N-acetylneuraminic acid hydroxylase occurred prior to brain expansion during human evolution. *Proceedings of the National Academy of Sciences of the United States of America* **99**, 11736-11741, doi:10.1073/pnas.182257399 (2002).

4 Zhang, Z. D., Frankish, A., Hunt, T., Harrow, J. & Gerstein, M. Identification and analysis of unitary pseudogenes: historic and contemporary gene losses in humans and other primates. *Genome Biol* **11**, R26, doi:10.1186/gb-2010-11-3-r26 (2010).

5 Hiller, M. *et al.* A "forward genomics" approach links genotype to phenotype using independent phenotypic losses among related species. *Cell Rep* **2**, 817-823, doi:10.1016/j.celrep.2012.08.032 (2012).

6 Prudent, X., Parra, G., Schwede, P., Roscito, J. G. & Hiller, M. Controlling for Phylogenetic Relatedness and Evolutionary Rates Improves the Discovery of Associations Between Species' Phenotypic and Genomic Differences. *Molecular biology and evolution* **33**, 2135-2150, doi:10.1093/molbev/msw098 (2016).

7 Oliveira, É. V. & Bergqvist, L. P. in *Paleógeno de América del Sur y de la Península Antártica* Vol. 5 (ed S Casadío) 35–40 (Publicación Especial, 1998).

8 Rosenbloom, K. R. *et al.* The UCSC Genome Browser database: 2015 update. *Nucleic Acids Res* **43**, D670-681, doi:10.1093/nar/gku1177 (2015).

9 Alvarez, E., Zhou, W., Witta, S. E. & Freed, C. R. Characterization of the Bex gene family in humans, mice, and rats. *Gene* **357**, 18-28, doi:10.1016/j.gene.2005.05.012 (2005).

10 Braun, B. A., Marcovitz, A., Camp, J. G., Jia, R. & Bejerano, G. Mx1 and Mx2 key antiviral proteins are surprisingly lost in toothed whales. *Proceedings of the National Academy of Sciences of the United States of America* **112**, 8036-8040, doi:10.1073/pnas.1501844112 (2015).

11 Cameron, J. *et al.* Investigations on the evolutionary conservation of PCSK9 reveal a functionally important protrusion. *FEBS J* **275**, 4121-4133, doi:10.1111/j.1742-4658.2008.06553.x (2008).

12 Derrien, T. *et al.* Revisiting the missing protein-coding gene catalog of the domestic dog. *BMC Genomics* **10**, 62, doi:10.1186/1471-2164-10-62 (2009).

13 Fang, X. *et al.* Genome-wide adaptive complexes to underground stresses in blind mole rats Spalax. *Nature communications* **5**, 3966, doi:10.1038/ncomms4966 (2014).

14 George, R. D. *et al.* Trans genomic capture and sequencing of primate exomes reveals new targets of positive selection. *Genome Res* **21**, 1686-1694, doi:10.1101/gr.121327.111 (2011).

15 He, J., Irwin, D. M., Chen, R. & Zhang, Y. P. Stepwise loss of motilin and its specific receptor genes in rodents. *J Mol Endocrinol* **44**, 37-44, doi:10.1677/JME-09-0095 (2010).

16 Jiang, P. *et al.* Major taste loss in carnivorous mammals. *Proceedings of the National Academy of Sciences of the United States of America* **109**, 4956-4961, doi:10.1073/pnas.1118360109 (2012).
17 Shen, B., Fang, T., Dai, M., Jones, G. & Zhang, S. Independent losses of visual perception genes Gja10 and Rbp3 in echolocating bats (Order: Chiroptera). *PloS one* **8**, e68867, doi:10.1371/journal.pone.0068867 (2013).
18 Strasser, B., Mlitz, V., Fischer, H., Tschachler, E. & Eckhart, L. Comparative genomics reveals conservation of filaggrin and loss of caspase-14 in dolphins. *Experimental dermatology* **24**, 365-369, doi:10.1111/exd.12681 (2015).
19 van der Loo, W. *et al.* Adaptive Gene Loss? Tracing Back the Pseudogenization of the Rabbit CCL8 Chemokine. *J Mol Evol* **83**, 12-25, doi:10.1007/s00239-016-9747-7 (2016).
20 Zhang, Q. *et al.* Characterization of AOC2 gene encoding a copper-binding amine oxidase expressed specifically in retina. *Gene* **318**, 45-53 (2003).
21 Meredith, R. W., Zhang, G., Gilbert, M. T., Jarvis, E. D. & Springer, M. S. Evidence for a single loss of mineralized teeth in the common avian ancestor. *Science* **346**, 1254390, doi:10.1126/science.1254390 (2014).
22 Springer, M. S. *et al.* Inactivation of C4orf26 in toothless placental mammals. *Mol Phylogenet Evol* **95**, 34-45, doi:10.1016/j.ympev.2015.11.002 (2016).
23 Reeb, D., Best, P. B. & Kidson, S. H. Structure of the integument of southern right whales, Eubalaena australis. *Anatomical record* **290**, 596-613, doi:10.1002/ar.20535 (2007).
24 Spearman, R. I. The epidermal stratum corneum of the whale. *Journal of anatomy* **113**, 373-381 (1972).
25 Mouton, M. & Botha, A. in *New Approaches to the Study of Marine Mammals* (eds A. Romero & E.O. Keith) (InTech, 2012).
26 Menon, G. K., Grayson, S., Brown, B. E. & Elias, P. M. Lipokeratinocytes of the epidermis of a cetacean (Phocena phocena). Histochemistry, ultrastructure, and lipid composition. *Cell Tissue Res* **244**, 385-394 (1986).
27 Menon, G. K. in *Skin, hair, and nails: structure and function* (ed L Norlén) (M. Dekker,, 2004).
28 Hicks, B. D., St Aubin, D. J., Geraci, J. R. & Brown, W. R. Epidermal growth in the bottlenose dolphin, Tursiops truncatus. *The Journal of investigative dermatology* **85**, 60-63 (1985).
29 Nery, M. F., Arroyo, J. I. & Opazo, J. C. Increased rate of hair keratin gene loss in the cetacean lineage. *BMC Genomics* **15**, 869, doi:10.1186/1471-2164-15-869 (2014).
30 Khan, I. *et al.* Mammalian keratin associated proteins (KRTAPs) subgenomes: disentangling hair diversity and adaptation to terrestrial and aquatic environments. *BMC Genomics* **15**, 779, doi:10.1186/1471-2164-15-779 (2014).
31 Chen, Z., Wang, Z., Xu, S., Zhou, K. & Yang, G. Characterization of hairless (Hr) and FGF5 genes provides insights into the molecular basis of hair loss in cetaceans. *BMC Evol Biol* **13**, 34, doi:10.1186/1471-2148-13-34 (2013).
32 Hoste, E. *et al.* Caspase-14-deficient mice are more prone to the development of parakeratosis. *The Journal of investigative dermatology* **133**, 742-750, doi:10.1038/jid.2012.350 (2013).
33 Bazzi, H. *et al.* Desmoglein 4 is expressed in highly differentiated keratinocytes and trichocytes in human epidermis and hair follicle. *Differentiation; research in biological diversity* **74**, 129-140, doi:10.1111/j.1432-0436.2006.00061.x (2006).

34 Descargues, P. *et al.* Corneodesmosomal cadherins are preferential targets of stratum corneum trypsin- and chymotrypsin-like hyperactivity in Netherton syndrome. *The Journal of investigative dermatology* **126**, 1622-1632, doi:10.1038/sj.jid.5700284 (2006).
35 Harrison, O. J. *et al.* Structural basis of adhesive binding by desmocollins and desmogleins. *Proceedings of the National Academy of Sciences of the United States of America* **113**, 7160-7165, doi:10.1073/pnas.1606272113 (2016).
36 Donetti, E. *et al.* Desmocollin 1 expression and desmosomal remodeling during terminal differentiation of human anagen hair follicle: an electron microscopic study. *Experimental dermatology* **13**, 289-297, doi:10.1111/j.0906-6705.2004.00152.x (2004).
37 Kljuic, A. *et al.* Desmoglein 4 in hair follicle differentiation and epidermal adhesion: evidence from inherited hypotrichosis and acquired pemphigus vulgaris. *Cell* **113**, 249-260 (2003).
38 Sundberg, J. P. *et al.* Lanceolate hair-J (lahJ): a mouse model for human hair disorders. *Experimental dermatology* **9**, 206-218 (2000).
39 Moss, C. *et al.* A recurrent intragenic deletion in the desmoglein 4 gene underlies localized autosomal recessive hypotrichosis. *The Journal of investigative dermatology* **123**, 607-610, doi:10.1111/j.0022-202X.2004.23311.x (2004).
40 John, P. *et al.* Recurrent intragenic deletion mutation in desmoglein 4 gene underlies autosomal recessive hypotrichosis in two Pakistani families of Balochi and Sindhi origins. *Archives of dermatological research* **298**, 135-137, doi:10.1007/s00403-006-0671-3 (2006).
41 Chidgey, M. *et al.* Mice lacking desmocollin 1 show epidermal fragility accompanied by barrier defects and abnormal differentiation. *J Cell Biol* **155**, 821-832, doi:10.1083/jcb.200105009 (2001).
42 Candi, E. *et al.* Expression of transglutaminase 5 in normal and pathologic human epidermis. *The Journal of investigative dermatology* **119**, 670-677, doi:10.1046/j.1523-1747.2002.01853.x (2002).
43 Candi, E. *et al.* Transglutaminase 5 cross-links loricrin, involucrin, and small proline-rich proteins in vitro. *J Biol Chem* **276**, 35014-35023, doi:10.1074/jbc.M010157200 (2001).
44 Cassidy, A. J. *et al.* A homozygous missense mutation in TGM5 abolishes epidermal transglutaminase 5 activity and causes acral peeling skin syndrome. *Am J Hum Genet* **77**, 909-917, doi:10.1086/497707 (2005).
45 Runkel, F. *et al.* The dominant alopecia phenotypes Bareskin, Rex-denuded, and Reduced Coat 2 are caused by mutations in gasdermin 3. *Genomics* **84**, 824-835, doi:10.1016/j.ygeno.2004.07.003 (2004).
46 Lunny, D. P. *et al.* Mutations in gasdermin 3 cause aberrant differentiation of the hair follicle and sebaceous gland. *The Journal of investigative dermatology* **124**, 615-621, doi:10.1111/j.0022-202X.2005.23623.x (2005).
47 Krieg, P. *et al.* Aloxe3 knockout mice reveal a function of epidermal lipoxygenase-3 as hepoxilin synthase and its pivotal role in barrier formation. *The Journal of investigative dermatology* **133**, 172-180, doi:10.1038/jid.2012.250 (2013).
48 Eckl, K. M. *et al.* Molecular analysis of 250 patients with autosomal recessive congenital ichthyosis: evidence for mutation hotspots in ALOXE3 and allelic heterogeneity in ALOX12B. *The Journal of investigative dermatology* **129**, 1421-1428, doi:10.1038/jid.2008.409 (2009).
49 Sokolov, V. E. *Mammal skin*. (Univ. of California Press, 1982).

50 Bossart, G. D., Meisner, R. A., Rommel, S. A., Ghim, S.-j. & Jenson, A. B. Pathological features of the Florida manatee cold stress syndrome. *Aquatic Mammals* **29**, 9-17 (2002).
51 Daniels, I. S., O. Brien WG, r., Nath, V., Zhao, Z. & Lee, C. C. AMP deaminase 3 deficiency enhanced 5'-AMP induction of hypometabolism. *PloS one* **8**, e75418, doi:10.1371/journal.pone.0075418 (2013).
52 O'Brien, W. G., 3rd, Berka, V., Tsai, A. L., Zhao, Z. & Lee, C. C. CD73 and AMPD3 deficiency enhance metabolic performance via erythrocyte ATP that decreases hemoglobin oxygen affinity. *Scientific reports* **5**, 13147, doi:10.1038/srep13147 (2015).
53 Cheng, J. *et al.* AMPD3-deficient mice exhibit increased erythrocyte ATP levels but anemia not improved due to PK deficiency. *Genes to cells : devoted to molecular & cellular mechanisms* **17**, 913-922, doi:10.1111/gtc.12006 (2012).
54 Ogasawara, N. *et al.* Deficiency of AMP deaminase in erythrocytes. *Human genetics* **75**, 15-18 (1987).
55 Ochiai, T., Goto, T. & Shikama, K. Effect of intracellular organic phosphates on the oxygen equilibrium curve of chicken hemoglobin. *Arch Biochem Biophys* **149**, 316-322 (1972).
56 Greaney, G. S., Hobish, M. K. & Powers, D. A. The effects of temperature and pH on the binding of ATP to carp (Cyprinus carpio) deoxyhemoglobin. *J Biol Chem* **255**, 445-453 (1980).
57 Watwood, S. L., Miller, P. J., Johnson, M., Madsen, P. T. & Tyack, P. L. Deep-diving foraging behaviour of sperm whales (Physeter macrocephalus). *J Anim Ecol* **75**, 814-825, doi:10.1111/j.1365-2656.2006.01101.x (2006).
58 Ponganis, P. J. Diving mammals. *Comprehensive Physiology* **1**, 447-465, doi:10.1002/cphy.c091003 (2011).
59 Storz, J. F. & Moriyama, H. Mechanisms of hemoglobin adaptation to high altitude hypoxia. *High Alt Med Biol* **9**, 148-157, doi:10.1089/ham.2007.1079 (2008).
60 Komiyama, N. H., Miyazaki, G., Tame, J. & Nagai, K. Transplanting a unique allosteric effect from crocodile into human haemoglobin. *Nature* **373**, 244-246, doi:10.1038/373244a0 (1995).
61 Bauer, C. & Jelkmann, W. Carbon dioxide governs the oxygen affinity of crocodile blood. *Nature* **269**, 825-827 (1977).
62 Bauer, C. *et al.* Analysis of bicarbonate binding to crocodilian hemoglobin. *J Biol Chem* **256**, 8429-8435 (1981).
63 Hessel, S. *et al.* CMO1 deficiency abolishes vitamin A production from beta-carotene and alters lipid metabolism in mice. *J Biol Chem* **282**, 33553-33561, doi:10.1074/jbc.M706763200 (2007).
64 Wald, G. Vitamins A in invertebrate eyes. *An,. Y. Physiol.* **153**, 479-480 (1941).
65 Brachi, R. M. Examination of some components of cephalopod and sperm-whale liver oils by the chromatographic method. *The Biochemical journal* **54**, 459-465 (1953).
66 Fisher, L. R., Kon, S. K. & Thompson, S. Y. Vitamin A and carotenoids in certain invertebrates. III. Euphausiacea. *Journal of the Marine Biological Association of the United Kingdom* **34**, 81-100, doi:DOI: http://dx.doi.org/10.1017/S0025315400008626 (1955).
67 Wagner, K. H. *Vitamin A and beta -carotene in the fin-back, blue and sperm whale*. (Johann Ambrosius Barth, Leipzig, 1939).

68 Kalko, E. K. V., Herre, E. A. & Handley, C. O. Relation of Fig Fruit Characteristics to Fruit-Eating Bats in the New and Old World Tropics. *Journal of Biogeography* **23**, 565-576 (1996).
69 Voigt, C. C. & Speakman, J. R. Nectar-feeding bats fuel their high metabolism directly with exogenous carbohydrates. *Functional Ecology* **21**, 913–921 (2007).
70 Arad, Z. & Korine, C. Effect of water restriction on energy and water balance and osmoregulation of the fruit bat Rousettus aegyptiacus. *Journal of comparative physiology. B, Biochemical, systemic, and environmental physiology* **163**, 401-405 (1993).
71 Happold, D. C. D. & Happold, M. Renal form and function in relation to the ecology of bats (Chiroptera) from Malawi, Central Africa. *Journal of zoology* **215**, 629–655 (1988).
72 Schondube, J. E., Herrera, M. L. & Martinez del Rio, C. Diet and the evolution of digestion and renal function in phyllostomid bats. *Zoology (Jena)* **104**, 59-73, doi:10.1078/0944-2006-00007 (2001).
73 Casotti, G., Gerardo Herrera, M. L., Flores, M. J., Mancina, C. A. & Braun, E. J. Relationships between renal morphology and diet in 26 species of new world bats (suborder microchiroptera). *Zoology (Jena)* **109**, 196-207, doi:10.1016/j.zool.2006.03.003 (2006).
74 Lopez-Nieto, C. E. *et al.* Molecular cloning and characterization of NKT, a gene product related to the organic cation transporter family that is almost exclusively expressed in the kidney. *J Biol Chem* **272**, 6471-6478 (1997).
75 Kojima, R. *et al.* Immunolocalization of multispecific organic anion transporters, OAT1, OAT2, and OAT3, in rat kidney. *Journal of the American Society of Nephrology : JASN* **13**, 848-857 (2002).
76 Motohashi, H. *et al.* Gene expression levels and immunolocalization of organic ion transporters in the human kidney. *Journal of the American Society of Nephrology : JASN* **13**, 866-874 (2002).
77 Eraly, S. A. *et al.* Decreased renal organic anion secretion and plasma accumulation of endogenous organic anions in OAT1 knock-out mice. *J Biol Chem* **281**, 5072-5083, doi:10.1074/jbc.M508050200 (2006).
78 Eraly, S. A. *et al.* Multiple organic anion transporters contribute to net renal excretion of uric acid. *Physiological genomics* **33**, 180-192, doi:10.1152/physiolgenomics.00207.2007 (2008).
79 Bahn, A. *et al.* Identification of a new urate and high affinity nicotinate transporter, hOAT10 (SLC22A13). *J Biol Chem* **283**, 16332-16341, doi:10.1074/jbc.M800737200 (2008).
80 Hagos, Y., Stein, D., Ugele, B., Burckhardt, G. & Bahn, A. Human renal organic anion transporter 4 operates as an asymmetric urate transporter. *Journal of the American Society of Nephrology : JASN* **18**, 430-439, doi:10.1681/ASN.2006040415 (2007).
81 Enomoto, A. *et al.* Molecular identification of a renal urate anion exchanger that regulates blood urate levels. *Nature* **417**, 447-452, doi:10.1038/nature742 (2002).
82 Augustin, R. *et al.* Identification and characterization of human glucose transporter-like protein-9 (GLUT9): alternative splicing alters trafficking. *J Biol Chem* **279**, 16229-16236, doi:10.1074/jbc.M312226200 (2004).
83 Vitart, V. *et al.* SLC2A9 is a newly identified urate transporter influencing serum urate concentration, urate excretion and gout. *Nat Genet* **40**, 437-442, doi:10.1038/ng.106 (2008).

84 Preitner, F. *et al.* Glut9 is a major regulator of urate homeostasis and its genetic inactivation induces hyperuricosuria and urate nephropathy. *Proceedings of the National Academy of Sciences of the United States of America* **106**, 15501-15506, doi:10.1073/pnas.0904411106 (2009).
85 Nakanishi, T., Ohya, K., Shimada, S., Anzai, N. & Tamai, I. Functional cooperation of URAT1 (SLC22A12) and URATv1 (SLC2A9) in renal reabsorption of urate. *Nephrol Dial Transplant* **28**, 603-611, doi:10.1093/ndt/gfs574 (2013).
86 Ludewig, U. Electroneutral ammonium transport by basolateral rhesus B glycoprotein. *The Journal of physiology* **559**, 751-759, doi:10.1113/jphysiol.2004.067728 (2004).
87 Verlander, J. W. *et al.* Localization of the ammonium transporter proteins RhBG and RhCG in mouse kidney. *Am J Physiol Renal Physiol* **284**, F323-337, doi:10.1152/ajprenal.00050.2002 (2003).
88 Bishop, J. M. *et al.* Role of the Rhesus glycoprotein, Rh B glycoprotein, in renal ammonia excretion. *Am J Physiol Renal Physiol* **299**, F1065-1077, doi:10.1152/ajprenal.00277.2010 (2010).
89 Lee, H. W. *et al.* Effect of dietary protein restriction on renal ammonia metabolism. *Am J Physiol Renal Physiol* **308**, F1463-1473, doi:10.1152/ajprenal.00077.2015 (2015).
90 Weiner, I. D. Roles of renal ammonia metabolism other than in acid-base homeostasis. *Pediatr Nephrol*, doi:10.1007/s00467-016-3401-x (2016).
91 Studier, E. H. & Wilson, D. E. Natural urine concentrations and composition in neotropical bats. *Comparative Biochemistry and Physiology Part A: Physiology* **75**, 509-515 (1983).
92 Yasui, M., Kwon, T. H., Knepper, M. A., Nielsen, S. & Agre, P. Aquaporin-6: An intracellular vesicle water channel protein in renal epithelia. *Proceedings of the National Academy of Sciences of the United States of America* **96**, 5808-5813 (1999).
93 Ikeda, M. *et al.* Characterization of aquaporin-6 as a nitrate channel in mammalian cells. Requirement of pore-lining residue threonine 63. *J Biol Chem* **277**, 39873-39879, doi:10.1074/jbc.M207008200 (2002).
94 Rambow, J., Wu, B., Ronfeldt, D. & Beitz, E. Aquaporins with anion/monocarboxylate permeability: mechanisms, relevance for pathogen-host interactions. *Frontiers in pharmacology* **5**, 199, doi:10.3389/fphar.2014.00199 (2014).
95 Liu, K. *et al.* Conversion of aquaporin 6 from an anion channel to a water-selective channel by a single amino acid substitution. *Proceedings of the National Academy of Sciences of the United States of America* **102**, 2192-2197, doi:10.1073/pnas.0409232102 (2005).
96 Promeneur, D. *et al.* Regulation of AQP6 mRNA and protein expression in rats in response to altered acid-base or water balance. *Am J Physiol Renal Physiol* **279**, F1014-1026 (2000).
97 Neuweiler, G. *Biology of Bats*. (Oxford University Press, 2000).
98 Voigt, C. C., Zubaid, A., Kunz, T. H. & Kingston, T. Sources of Assimilated Proteins in Old and New World Phytophagous Bats. *Biotropica* **43**, 108–113 (2010).
99 Amitai, O. *et al.* Fruit bats (Pteropodidae) fuel their metabolism rapidly and directly with exogenous sugars. *The Journal of experimental biology* **213**, 2693-2699, doi:10.1242/jeb.043505 (2010).
100 Welch, K. C., Jr., Herrera, M. L. & Suarez, R. K. Dietary sugar as a direct fuel for flight in the nectarivorous bat Glossophaga soricina. *The Journal of experimental biology* **211**, 310-316, doi:10.1242/jeb.012252 (2008).

101 Yen, C. L. *et al.* Deficiency of the intestinal enzyme acyl CoA:monoacylglycerol acyltransferase-2 protects mice from metabolic disorders induced by high-fat feeding. *Nat Med* **15**, 442-446, doi:10.1038/nm.1937 (2009).
102 Yen, C. L. & Farese, R. V., Jr. MGAT2, a monoacylglycerol acyltransferase expressed in the small intestine. *J Biol Chem* **278**, 18532-18537, doi:10.1074/jbc.M301633200 (2003).
103 Agellon, L. B., Toth, M. J. & Thomson, A. B. Intracellular lipid binding proteins of the small intestine. *Molecular and cellular biochemistry* **239**, 79-82 (2002).
104 Praslickova, D. *et al.* The ileal lipid binding protein is required for efficient absorption and transport of bile acids in the distal portion of the murine small intestine. *PloS one* **7**, e50810, doi:10.1371/journal.pone.0050810 (2012).
105 Gimeno, R. E. *et al.* Characterization of a heart-specific fatty acid transport protein. *J Biol Chem* **278**, 16039-16044, doi:10.1074/jbc.M211412200 (2003).
106 Bing, R. Myocardial metabolism. *Circulation* **12**, 635-647 (1955).
107 Albrektsen, T., Richter, H. E., Clausen, J. T. & Fleckner, J. Identification of a novel integral plasma membrane protein induced during adipocyte differentiation. *The Biochemical journal* **359**, 393-402 (2001).
108 Bogner-Strauss, J. G. *et al.* Reconstruction of gene association network reveals a transmembrane protein required for adipogenesis and targeted by PPARgamma. *Cell Mol Life Sci* **67**, 4049-4064, doi:10.1007/s00018-010-0424-5 (2010).
109 Welch, K. C., Jr., Peronnet, F., Hatch, K. A., Voigt, C. C. & McCue, M. D. Carbon stable-isotope tracking in breath for comparative studies of fuel use. *Ann N Y Acad Sci* **1365**, 15-32, doi:10.1111/nyas.12737 (2016).
110 Voigt, C. C., Sorgel, K., Suba, J., Keiss, O. & Petersons, G. The insectivorous bat Pipistrellus nathusii uses a mixed-fuel strategy to power autumn migration. *Proceedings. Biological sciences / The Royal Society* **279**, 3772-3778, doi:10.1098/rspb.2012.0902 (2012).
111 O'Mara, M. T. *et al.* Cyclic bouts of extreme bradycardia counteract the high metabolism of frugivorous bats. *eLife* **6**, doi:10.7554/eLife.26686 (2017).
112 Wang, O. *et al.* Mechanisms of glucose-induced expression of pancreatic-derived factor in pancreatic beta-cells. *Endocrinology* **149**, 672-680, doi:10.1210/en.2007-0106 (2008).
113 Yang, J. *et al.* Mechanisms of glucose-induced secretion of pancreatic-derived factor (PANDER or FAM3B) in pancreatic beta-cells. *Diabetes* **54**, 3217-3228 (2005).
114 Robert-Cooperman, C. E. *et al.* PANDER transgenic mice display fasting hyperglycemia and hepatic insulin resistance. *The Journal of endocrinology* **220**, 219-231, doi:10.1530/joe-13-0338 (2014).
115 Moak, S. L. *et al.* Enhanced glucose tolerance in pancreatic-derived factor (PANDER) knockout C57BL/6 mice. *Disease models & mechanisms* **7**, 1307-1315, doi:10.1242/dmm.016402 (2014).
116 Tang, C. *et al.* Loss of FFA2 and FFA3 increases insulin secretion and improves glucose tolerance in type 2 diabetes. *Nat Med* **21**, 173-177, doi:10.1038/nm.3779 (2015).
117 Priyadarshini, M. & Layden, B. T. FFAR3 modulates insulin secretion and global gene expression in mouse islets. *Islets* **7**, e1045182, doi:10.1080/19382014.2015.1045182 (2015).
118 Protzek, A. O. *et al.* Insulin and glucose sensitivity, insulin secretion and beta-cell distribution in endocrine pancreas of the fruit bat Artibeus lituratus. *Comparative*

biochemistry and physiology. Part A, Molecular & integrative physiology **157**, 142-148, doi:10.1016/j.cbpa.2010.05.016 (2010).

119 Lambert, O. *et al.* The giant bite of a new raptorial sperm whale from the Miocene epoch of Peru. *Nature* **466**, 105-108, doi:10.1038/nature09067 (2010).

120 Meredith, R. W., Gatesy, J., Cheng, J. & Springer, M. S. Pseudogenization of the tooth gene enamelysin (MMP20) in the common ancestor of extant baleen whales. *Proceedings. Biological sciences / The Royal Society* **278**, 993-1002, doi:10.1098/rspb.2010.1280 (2011).

121 Parry, D. A. *et al.* Mutations in C4orf26, encoding a peptide with in vitro hydroxyapatite crystal nucleation and growth activity, cause amelogenesis imperfecta. *Am J Hum Genet* **91**, 565-571, doi:10.1016/j.ajhg.2012.07.020 (2012).

122 Seymen, F. *et al.* Recessive Mutations in ACPT, Encoding Testicular Acid Phosphatase, Cause Hypoplastic Amelogenesis Imperfecta. *Am J Hum Genet* **99**, 1199-1205, doi:10.1016/j.ajhg.2016.09.018 (2016).

123 Yang, W. *et al.* Natural flexible dermal armor. *Adv Mater* **25**, 31-48, doi:10.1002/adma.201202713 (2013).

124 Scrima, A. *et al.* Structural basis of UV DNA-damage recognition by the DDB1-DDB2 complex. *Cell* **135**, 1213-1223, doi:10.1016/j.cell.2008.10.045 (2008).

125 Yeh, J. I. *et al.* Damaged DNA induced UV-damaged DNA-binding protein (UV-DDB) dimerization and its roles in chromatinized DNA repair. *Proceedings of the National Academy of Sciences of the United States of America* **109**, E2737-2746, doi:10.1073/pnas.1110067109 (2012).

126 Rapic-Otrin, V. *et al.* True XP group E patients have a defective UV-damaged DNA binding protein complex and mutations in DDB2 which reveal the functional domains of its p48 product. *Human molecular genetics* **12**, 1507-1522 (2003).

127 Itoh, T., Cado, D., Kamide, R. & Linn, S. DDB2 gene disruption leads to skin tumors and resistance to apoptosis after exposure to ultraviolet light but not a chemical carcinogen. *Proceedings of the National Academy of Sciences of the United States of America* **101**, 2052-2057, doi:10.1073/pnas.0306551101 (2004).

128 Yoon, T. *et al.* Tumor-prone phenotype of the DDB2-deficient mice. *Oncogene* **24**, 469-478, doi:10.1038/sj.onc.1208211 (2005).

129 von Koenigswald, W., Richter, G. & Storch, G. Nachweis von Hornschuppen bei Eomanis waldi aus der "Grube Messe!" bei Darmstadt (Mammalia, Pholidota). *Senckenbergiana lethaea* **61**, 291-298 (1981).

130 Szklarczyk, D. *et al.* The STRING database in 2017: quality-controlled protein-protein association networks, made broadly accessible. *Nucleic Acids Res* **45**, D362-D368, doi:10.1093/nar/gkw937 (2017).

131 Marteijn, J. A., Lans, H., Vermeulen, W. & Hoeijmakers, J. H. Understanding nucleotide excision repair and its roles in cancer and ageing. *Nat Rev Mol Cell Biol* **15**, 465-481, doi:10.1038/nrm3822 (2014).

132 Gao, J. *et al.* The CUL4-DDB1 ubiquitin ligase complex controls adult and embryonic stem cell differentiation and homeostasis. *eLife* **4**, doi:10.7554/eLife.07539 (2015).

133 Ho, J. J., Cattoglio, C., McSwiggen, D. T., Tjian, R. & Fong, Y. W. Regulation of DNA demethylation by the XPC DNA repair complex in somatic and pluripotent stem cells. *Genes Dev* **31**, 830-844, doi:10.1101/gad.295741.116 (2017).

134 Németh, A. *et al.* Danger underground and in the open – predation on blind mole rats (Rodentia: Spalacinae) revisited. *Mammal Review* **46**, 204-214, doi:10.1111/mam.12062 (2016).
135 Hecker, N., Sharma, V. & Hiller, M. Transition to an Aquatic Habitat Permitted the Repeated Loss of the Pleiotropic KLK8 Gene in Mammals. *Genome Biol Evol* **9**, 3179-3188, doi:10.1093/gbe/evx239 (2017).
136 Shipley, J. M., Wesselschmidt, R. L., Kobayashi, D. K., Ley, T. J. & Shapiro, S. D. Metalloelastase is required for macrophage-mediated proteolysis and matrix invasion in mice. *Proceedings of the National Academy of Sciences of the United States of America* **93**, 3942-3946 (1996).
137 Marchant, D. J. *et al.* A new transcriptional role for matrix metalloproteinase-12 in antiviral immunity. *Nat Med* **20**, 493-502, doi:10.1038/nm.3508 (2014).
138 Matsumoto, S. *et al.* Expression and localization of matrix metalloproteinase-12 in the aorta of cholesterol-fed rabbits: relationship to lesion development. *Am J Pathol* **153**, 109-119 (1998).
139 Pellicoro, A. *et al.* Elastin accumulation is regulated at the level of degradation by macrophage metalloelastase (MMP-12) during experimental liver fibrosis. *Hepatology* **55**, 1965-1975, doi:10.1002/hep.25567 (2012).
140 Chung, A. W. *et al.* Matrix metalloproteinase-2 and -9 exacerbate arterial stiffening and angiogenesis in diabetes and chronic kidney disease. *Cardiovasc Res* **84**, 494-504, doi:10.1093/cvr/cvp242 (2009).
141 Liu, S. L. *et al.* Matrix metalloproteinase-12 is an essential mediator of acute and chronic arterial stiffening. *Scientific reports* **5**, 17189, doi:10.1038/srep17189 (2015).
142 Skjot-Arkil, H. *et al.* Measurement of MMP-9 and -12 degraded elastin (ELM) provides unique information on lung tissue degradation. *BMC Pulm Med* **12**, 34, doi:10.1186/1471-2466-12-34 (2012).
143 Houghton, A. M. Matrix metalloproteinases in destructive lung disease. *Matrix Biol* **44-46**, 167-174, doi:10.1016/j.matbio.2015.02.002 (2015).
144 Hautamaki, R. D., Kobayashi, D. K., Senior, R. M. & Shapiro, S. D. Requirement for macrophage elastase for cigarette smoke-induced emphysema in mice. *Science* **277**, 2002-2004 (1997).
145 Houghton, A. M. *et al.* Elastin fragments drive disease progression in a murine model of emphysema. *The Journal of clinical investigation* **116**, 753-759, doi:10.1172/JCI25617 (2006).
146 Hunninghake, G. M. *et al.* MMP12, lung function, and COPD in high-risk populations. *N Engl J Med* **361**, 2599-2608, doi:10.1056/NEJMoa0904006 (2009).
147 Kooyman, G. L. & Cornell, L. H. Flow Properties of Expiration and Inspiration in a Trained Bottle-Nosed Porpoise. *Physiological Zoology* **54**, 55-61 (1981).
148 Berta, A., Sumich, J. L., Kovacs, K. M., Folkens, P. A. & Adam, P. J. in *Marine Mammals (Second Edition)* 237-269 (Academic Press, 2006).
149 Piscitelli, M. A., Raverty, S. A., Lillie, M. A. & Shadwick, R. E. A review of cetacean lung morphology and mechanics. *J Morphol* **274**, 1425-1440, doi:10.1002/jmor.20192 (2013).
150 Vennell, R., Pease, D. & Wilson, B. Wave drag on human swimmers. *J Biomech* **39**, 664-671, doi:10.1016/j.jbiomech.2005.01.023 (2006).
