# Supplementary material for: A genomics approach reveals insights into the importance of gene losses for mammalian adaptations
Source: Nat Commun. 2018 Mar 23;9:1215. doi: 10.1038/s41467-018-03667-1 (PMC5865188; doi:10.1038/s41467-018-03667-1)
Supplement: Supplementary file 2 — Description of Additional Supplementary Files(PDF 167 kb) [file 41467_2018_3667_MOESM2_ESM.pdf]

Description of Additional Supplementary Files

File Name: Supplementary Data 1
Description: 13,486 genes that are 1:1 orthologs between human and mouse/rat/dog/cow, and are thus most likely truly conserved.
